# Genome Diversity, Recombination, and Virulence across the Major Lineages of *Paracoccidioides*

**DOI:** 10.1128/mSphere.00213-16

**Published:** 2016-09-28

**Authors:** José F. Muñoz, Rhys A. Farrer, Christopher A. Desjardins, Juan E. Gallo, Sean Sykes, Sharadha Sakthikumar, Elizabeth Misas, Emily A. Whiston, Eduardo Bagagli, Celia M. A. Soares, Marcus de M. Teixeira, John W. Taylor, Oliver K. Clay, Juan G. McEwen, Christina A. Cuomo

**Affiliations:** a Cellular and Molecular Biology Unit, Corporación para Investigaciones Biológicas, Medellín, Colombia; b Institute of Biology, Universidad de Antioquia, Medellín, Colombia; c Broad Institute of MIT and Harvard, Cambridge, Massachusetts, USA; d Doctoral Program in Biomedical Sciences, Universidad del Rosario, Bogotá, Colombia; e Department of Plant and Microbial Biology, University of California, Berkeley, Berkeley, California, USA; f Instituto de Biociências, Universidade Estadual Paulista, Botucatu, São Paulo, Brazil; g Laboratório de Biología Molecular, Instituto de Ciências Biológicas, ICBII, Goiânia, Brazil; h Instituto de Ciências Biológicas, Universidade de Brasília, Brasília, Distrito Federal, Brazil; i Division of Pathogen Genomics, Translational Genomics Research Institute North, Flagstaff, Arizona, USA; j School of Medicine and Health Sciences, Universidad del Rosario, Bogotá, Colombia; k School of Medicine, Universidad de Antioquia, Medellín, Colombia; Carnegie Mellon University

**Keywords:** *Paracoccidioides*, evolution, genetic recombination, genome analysis, mycology, population genetics

## Abstract

The *Paracoccidioides* genus includes two species of thermally dimorphic fungi that cause paracoccidioidomycosis, a neglected health-threatening human systemic mycosis endemic to Latin America. To examine the genome evolution and the diversity of *Paracoccidioides* spp., we conducted whole-genome sequencing of 31 isolates representing the phylogenetic, geographic, and ecological breadth of the genus. These samples included clinical, environmental and laboratory reference strains of the S1, PS2, PS3, and PS4 lineages of *P. brasiliensis* and also isolates of *Paracoccidioides lutzii* species*.* We completed the first annotated genome assemblies for the PS3 and PS4 lineages and found that gene order was highly conserved across the major lineages, with only a few chromosomal rearrangements*.* Comparing whole-genome assemblies of the major lineages with single-nucleotide polymorphisms (SNPs) predicted from the remaining 26 isolates, we identified a deep split of the S1 lineage into two clades we named S1a and S1b. We found evidence for greater genetic exchange between the S1b lineage and all other lineages; this may reflect the broad geographic range of S1b, which is often sympatric with the remaining, largely geographically isolated lineages. In addition, we found evidence of positive selection for the *GP43* and *PGA1* antigen genes and genes coding for other secreted proteins and proteases and lineage-specific loss-of-function mutations in cell wall and protease genes; these together may contribute to virulence and host immune response variation among natural isolates of *Paracoccidioides* spp. These insights into the recent evolutionary events highlight important differences between the lineages that could impact the distribution, pathogenicity, and ecology of *Paracoccidioides*.

**IMPORTANCE** Characterization of genetic differences between lineages of the dimorphic human-pathogenic fungus *Paracoccidioides* can identify changes linked to important phenotypes and guide the development of new diagnostics and treatments. In this article, we compared genomes of 31 diverse isolates representing the major lineages of *Paracoccidioides* spp. and completed the first annotated genome sequences for the PS3 and PS4 lineages. We analyzed the population structure and characterized the genetic diversity among the lineages of *Paracoccidioides*, including a deep split of S1 into two lineages (S1a and S1b), and differentiated S1b, associated with most clinical cases, as the more highly recombining and diverse lineage. In addition, we found patterns of positive selection in surface proteins and secreted enzymes among the lineages, suggesting diversifying mechanisms of pathogenicity and adaptation across this species complex. These genetic differences suggest associations with the geographic range, pathogenicity, and ecological niches of *Paracoccidioides* lineages.

## Abstract (precis)

Characterization of genetic differences between lineages of the dimorphic human-pathogenic fungus *Paracoccidioides* can identify changes linked to important phenotypes and guide the development of new diagnostics and treatments. In this article, we compared genomes of 31 diverse isolates representing the major lineages of *Paracoccidioides* spp. and completed the first annotated genome sequences for the PS3 and PS4 lineages. We analyzed the population structure and characterized the genetic diversity among the lineages of *Paracoccidioides*, including a deep split of S1 into two lineages (S1a and S1b), and differentiated S1b, associated with most clinical cases, as the more highly recombining and diverse lineage. In addition, we found patterns of positive selection in surface proteins and secreted enzymes among the lineages, suggesting diversifying mechanisms of pathogenicity and adaptation across this species complex. These genetic differences suggest associations with the geographic range, pathogenicity, and ecological niches of *Paracoccidioides* lineages.

## INTRODUCTION

*Paracoccidioides* spp. are the cause of paracoccidioidomycosis (PCM), a systemic mycosis that mainly affects people in Latin America. In this region where PCM is endemic, PCM has an estimated incidence of 1 to 3 cases per 100,000 inhabitants (1, 2). The vast majority of PCM cases (roughly 80%) occur in Brazil, while Colombia and Venezuela have the next highest numbers of infections (3). *Paracoccidioides* is a thermally dimorphic fungus closely related to *Histoplasma* and *Blastomyces*, which cause similar infections worldwide or predominantly in regions of North America, respectively.

Multilocus sequencing studies elucidated species boundaries within the *Paracoccidioides* genus and supported the existence of two distinct species, *P. brasiliensis* and *P. lutzii* (4). *P. lutzii* is a single monophyletic and recombining population found to date in central, southwest, and north Brazil and Ecuador (4). *P. brasiliensis* is monophyletic and is comprised of distinct lineages classified as S1, PS2, PS3, and PS4 (4–6). The S1 lineage is associated with the majority of PCM cases and is widely distributed in South America (4–6). PS2 has been identified to date only in Brazil and Venezuela, whereas PS3 is mainly found in regions of endemicity in Colombia (4, 5). Recently, a novel lineage, PS4, was described from a region of Venezuela (6). Evidence of recombination was noted for *P. brasiliensis* S1 and *P. lutzii*, but not other lineages, based on a small number of genomic loci (4, 5).

Isolates from each of these phylogenetic lineages of *Paracoccidioides* can infect humans; however, different lineages can vary in virulence and culture adaptation and can induce different immune responses by the host (7, 8). One feature that is correlated with the differential rates of infection is variation in the number of infective conidia. For example, isolates from S1 produce many more conidia than PS2 isolates, which could be related to the disproportional 9:1 rate of S1 to PS2 infection in both human and armadillo isolates (8). In addition to interspecific variation between lineages and between species, *Paracoccidioides* isolates have been shown to contain extensive intraspecific genetic variability between strains of the same lineage (9–11).

To enable genome-based studies of this medically important fungus, isolates of *P. brasiliensis* S1 and PS2 and *P. lutzii* were previously sequenced and compared to related dimorphic and nondimorphic fungi (12). Notably, *Paracoccidioides* and related dimorphic pathogens have a reduced number of genes involved in carbohydrate metabolism, protein metabolism, and synthesis of secondary metabolites (12), an observation that allows new insights into the differences between these related fungi and their physiological potential for pathogenicity. Recently, the genome assemblies and gene annotations of those reference strains were improved using Illumina resequencing, increasing the overall accuracy of assembly bases and gene structures (13). These improved reference genomes of *Paracoccidioides* spp. provide an opportunity to map the population structure and examine variation with finer resolution.

In this study, we used genome sequences of 31 isolates for a comprehensive comparison of gene conservation, genetic diversity, and genome evolution across the major lineages of *Paracoccidioides*. The panel of isolates sequenced in this study included clinical isolates from acute and chronic PCM cases and environmental isolates from soil or two species of armadillos (*Dasypus novemcinctus* and *Cabassous centralis*). We assembled the first reference genomes for the PS3 and PS4 lineages: compared to the previously assembled references from other lineages, gene content and order are highly conserved, with few rearrangements. We characterized genetic diversity among the lineages, as well as lineage-specific evolutionary patterns within the *Paracoccidioides* genus and found evidence of recombination and ancestral hybridization patterns between some of the lineages. Additionally, we identified genomic regions or genes that are highly diverse within or between lineages; these include genes with potential roles in virulence. We found that genes with the strongest evidence of positive selection include the *GP43* antigen gene and genes coding for other secreted proteins and proteases (e.g., *PGA1*, *CBP1*, *SOD3*, and *ENG1*), as well as loss-of-function mutation in genes that are specific to some lineages. Our analyses provide insight into the recent evolutionary events highlighting genetic differences between the lineages that could impact the distribution, pathogenicity, and ecology of *Paracoccidioides*. These potential virulence factors and genetic differences at the population level will be important for future studies to compare with the infectious potency of *Paracoccidioides* clinical isolates and clinical symptoms attributable to paracoccidioidomycosis.

## RESULTS

### Conserved genome organization across *Paracoccidioides* spp.

To compare genome structures and gene contents between the major *Paracoccidioides* lineages, we sequenced, assembled, and annotated a representative isolate from the PS3 lineage (strain PbCnh) and from the PS4 lineages (strain Pb300) (see Fig. S1A and Text S1 in the supplemental material). The 29.4-Mb assemblies of both strains are intermediate in size between those of the 29.95-Mb assembly of Pb18 (S1b) and the 29.06-Mb assembly of Pb03 (PS2) (see Fig. S1B) (13). The gene annotation of strains PbCnh and Pb300 resulted in 8,324 and 8,070 predicted protein-coding genes, respectively. High representation of core eukaryotic genes provides evidence that those genomes are nearly complete; 96 to 98% of these conserved genes are found in all assemblies (see Fig. S1C). Predicted gene contents were highly similar across all four *P. brasiliensis* genomes, comparing the new assemblies to the previously sequenced genomes (see Fig. S1B), which suggests that the gene content is very consistent across the lineages.

10.1128/mSphere.00213-16.1 Text S1  The genomes of the *P. brasiliensis* PS3 and PS4 lineages, gene conservation in *Paracoccidioides*, and candidate genes with experimental evidence for a role in virulence and pathogenicity. Download Text S1, DOCX file, 0.1 MB. Copyright © 2016 Muñoz et al. 2016 Muñoz et al. This content is distributed under the terms of the Creative Commons Attribution 4.0 International license.

10.1128/mSphere.00213-16.3 Figure S1  Assembly and annotation statistics for the PS3 and PS4 reference genomes and synteny of *Paracoccidioides* genomes. (A) Basic assembly statistics of PbCnh (PS3) and Pb300 (PS4). (B) Genome size and gene counts of PbCnh (PS3) and Pb300 (PS4) (blue bars) compared with other reference genomes of *Paracoccidioides* (gray bars). (C) Conservation of the core eukaryotic gene (CEG) set is similar in PbCnh, Pb300, and other *Paracoccidioides* genomes. (D) Nucmer alignments of *Paracoccidioides* genomes and average percentage of identity for each comparison. (E) Number of syntenic blocks and percentage of genes in syntenic blocks of *Paracoccidioides* genomes based on DAGchainer. (F) Synteny of four reference genomes of *Paracoccidioides* representing major lineages. Shown is a visualization of the synteny (gray) and structural variants between representatives for each lineage: S1 (Pb18), PS2 (Pb03), PS3 (PbCnh), and *P. lutzii* (Pb01). Genes in regions that have undergone chromosomal rearrangements are shown as blue dots and *MAT* locus genes as green dots, and some known virulence factors and genes under positive selection are shown as red dots. Scaffold numbers are shown along with orientation (±). Download Figure S1, PDF file, 1.9 MB. Copyright © 2016 Muñoz et al. 2016 Muñoz et al. This content is distributed under the terms of the Creative Commons Attribution 4.0 International license.

The *Paracoccidioides* genomes of both species and all lineages are highly conserved in terms of whole-genome sequence similarity and gene synteny (see Text S1 and Fig. S1D). The genomes of *P. brasiliensis* share an average of 98.5% identity, whereas the genome of the more distant species *P. lutzii* shares an average of 94.8% with *P. brasiliensis*. The genomes of PbCnh (PS3) and Pb300 (PS4) share the highest percentage aligned (98.9%), which correlates with the phylogenetic and population structure relationships (see below). We identified syntenic regions of conserved gene order and found an average of 6,907 genes within syntenic blocks among the *Paracoccidioides* lineages (see Fig. S1D to F). The percentage of genes in syntenic blocks ranged from 75.3% (interspecies, *P. brasiliensis* versus *P. lutzii*) to 89.9% (intraspecies, *P. brasiliensis* versus *P. brasiliensis*). In contrast, the dimorphic fungus *Blastomyces* has only ~69% genes in syntenic blocks due to the presence of isochore-like structures of repeat-rich GC-poor and GC-rich blocks rarely observed in *Paracoccidioides* (14).

While the *Paracoccidioides* genomes were largely colinear, a few chromosomal rearrangements were detected. A large rearrangement was detected between *P. brasiliensis* S1/PS3 and *P. lutzii*/*P. brasiliensis* PS2 in chromosome 4, where the regions at the beginning and the end of the supercontig 4 are inverted, and the gene order across the middle of the supercontig is conserved (see Fig. S1F). A large chromosomal rearrangement was also detected between *P. brasiliensis* and *P. lutzii*, where syntenic blocks in *P. brasiliensis* chromosomes 3 and 5 (Pb18, supercontigs 2, 13, and 10) are combined into a single supercontig in *P. lutzii* (see Fig. S1F). We found no evidence of assembly errors across the junctions of these rearrangements based on even coverage of aligned reads across these regions. In addition, we called structural variants based on the read alignments to the Pb18 assembly, and recovered each of the rearrangements present in the assemblies (see Text S1  and Data Set S1 in the supplemental material). Chromosomal rearrangements may impact the capacity for genetic exchange, as some crossover events will generate missing chromosomal regions or other aneuploidies and nonviable progeny. The rearrangement between PS2 and the other lineages of *P. brasiliensis* could potentially prevent genetic exchange between these groups.

10.1128/mSphere.00213-16.2 Data Set S1  (Tab 1) Full functional and genetic annotation of *Paracoccidioides* genes, including details of genes located in windows where SNP density, nucleotide diversity, and Tajima’s *D* were more than 2 standard deviations from the mean, where the *F_ST_* was less than 2 standard deviations from the mean, and genes evolved under positive selection (*dN*/*dS* of >1). (Tab 2) Numbers of synonymous and nonsynonymous mutations for virulence-associated or yeast-phase-specific genes in *Paracoccidioides* or other dimorphic fungi. (Tab 3) List of unique genes in *Paracoccidioides* reference genomes according to ortholog cluster analysis. (Tab 4) Predicted chromosomal structural variants (>10 kb) using BreakDancer in *Paracoccidioides* reference genomes. Download Data Set S1, XLSX file, 0.6 MB. Copyright © 2016 Muñoz et al. 2016 Muñoz et al. This content is distributed under the terms of the Creative Commons Attribution 4.0 International license.

In addition to chromosomal rearrangements, we looked for evidence of copy number and ploidy variation. We calculated the normalized read alignment density in 10-kb nonoverlapping windows (see Materials and Methods) and examined the variation across the Pb18 chromosomes. The alignment density showed no large regions of higher sequencing depth, supporting that *Paracoccidioides* species do not maintain aneuploid chromosomes or segments (see Fig. S2 in the supplemental material) such as those found in other fungi.

10.1128/mSphere.00213-16.4 Figure S2  Lack of large duplicated regions or aneuploidies based on normalized read density and SNP density distribution. (A) Each panel corresponds to one strain, labeled at the left. The normalized sequence depth (*y* axis) centered on 1× is shown across the genome assembly of Pb18 reference (*x* axis). Only small regions of coverage variation are observed, including the small more repetitive scaffolds. (B) For each strain listed along the *y* axis, the SNP density (number of SNPs/kilobase) is plotted across the Pb18 reference. Lineage groupings are noted by different colors, corresponding to the groups listed at the right. Download Figure S2, PDF file, 2.1 MB. Copyright © 2016 Muñoz et al. 2016 Muñoz et al. This content is distributed under the terms of the Creative Commons Attribution 4.0 International license.

### Phylogenetic for S1 group split and allele sharing between lineages.

**FIG 1:**
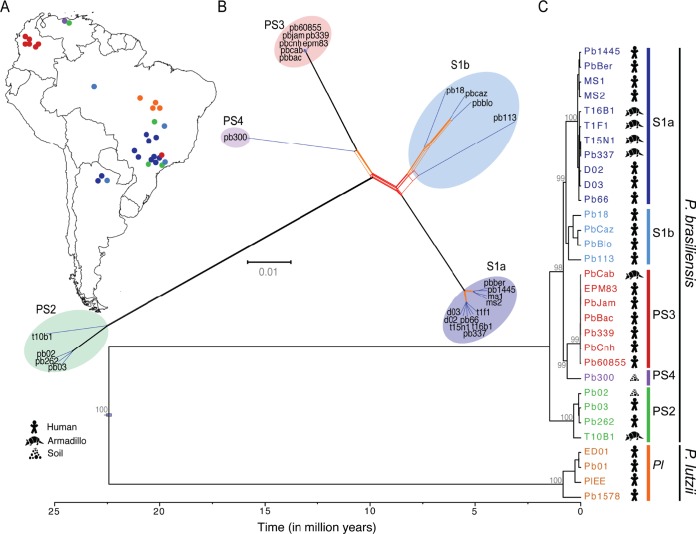
Phylogeny and recombination in *Paracoccidioides*. Two methods were used to examine strain relationships originating from across South America (A): using 614,570 SNPs, including a phylogenetic network constructed with SplitsTree4 (B), and a Bayesian calibrated phylogeny constructed with BEAST (C); bootstrap values from maximum likelihood phylogeny constructed with RAxML were included for major subdivisions. Both methods show evidence of five distinct lineages in *P. brasiliensis*: S1 (blue), which is divided into two groups S1a (dark blue) and S1b (light blue), PS2 (green), PS3 (red), and the recently described PS4 (purple). Also, this phylogeny supports the divergence between *P. brasiliensis* and *P. lutzii* (*Pl* [orange]) as a different species. In addition, the phylogenetic network of *P. brasiliensis* suggests patterns of recombination (red branches).

To examine the *Paracoccidioides* phylogenetic relationships, we identified polymorphisms across an extended panel of 31 isolates (Table 1). Using 614,570 positions (single-nucleotide polymorphisms [SNPs]) in the sequenced isolates, maximum likelihood and Bayesian phylogenies were constructed to examine intralineage relationships (Materials and Methods). Both maximum likelihood and Bayesian analyses highly supported that *P. brasiliensis* isolates are clustered into five distinct lineages (Fig. 1; see Fig. S3 in the supplemental material). Of the four previously identified lineages, S1 is the most highly variable, with two distinct clades we denoted as the S1a and S1b lineages. The S1b lineage includes the reference strain Pb18, along with three clinical isolates from Brazil and Argentina. The S1a lineage includes clinical and environmental isolates and was split into two subclades: one includes clinical isolates from Argentina and central-west regions of Brazil, while the second includes three clinical and four armadillo isolates from southeast Brazil. This phylogenetic analysis also supports that PS3 is a monophyletic group with very limited diversity of mostly isolates from Colombia, including both chronic PCM isolates and one isolate from armadillo. While previous phylogenies using short loci had suggested that PS3 only includes isolates from Colombia (4, 5, 8), we found that the Pb339 isolate from southeast Brazil was placed in this lineage (Fig. 1), suggesting that this lineage may be more widespread than previously described. In addition, our phylogenetic analysis provides strong evidence for the separation of the PS4 lineage, as recently proposed (6), sharing a common ancestor with PS3. This was also supported by population structure analyses (see below). To confirm that the phylogeny was not influenced by the use of a reference genome from one lineage, we identified SNPs from reads aligned to reference genomes representing each lineage (Pb03, PbCnh, Pb300, and Pb01) and found the same topology in phylogenies of each set (see Fig. S3).

10.1128/mSphere.00213-16.5 Figure S3  Whole-genome phylogenies of SNPs mapped against each reference genome representing each lineage. Each maximum likelihood phylogeny supports the same subdivision of *P. brasiliensis* in distinct lineages: S1 (blue), which is clearly split into two subgroups, S1a and S1b; PS2 (green); PS3 (red); and the recently described PS4 (purple). These phylogenies also support the separation of *P. brasiliensis* and *P. lutzii* (Pl [orange]) as different species. Download Figure S3, PDF file, 0.5 MB. Copyright © 2016 Muñoz et al. 2016 Muñoz et al. This content is distributed under the terms of the Creative Commons Attribution 4.0 International license.

**TABLE 1:** *Paracoccidioides* species isolates selected for this study

Isolate ID	Other name(s)	Origin	Source	Lineage	Provider	Reference
*P. brasiliensis*						
Pb18^a^	B17	Sao Paulo, Brazil	Chronic PCM^b^	S1b	R. Puccia	44
PbCaz	Cazon; A1	Chaco, Argentina	Acute PCM	S1b	R. Negroni	45
Pb113		Manaus-AM, Brazil	PCM	S1b	C. de Almeida Soares	46
PbBlo		Brazil	PCM	S1b	C. de Almeida Soares	This study
MS1		Mato Grosso do Sul, Brazil	PCM	S1a	M. Sueli Felipe	47
D03		Piracicaba, SP, Brazil	PCM	S1a	E. Bagagli	This study
MS2		Mato Grosso do Sul, Brazil	PCM	S1a	M. Sueli Felipe	47
Pb1445	A5	Argentina	Chronic PCM	S1a	R. Negroni	5
Pb337	T15LN1; B10	Brazil	*D. novemcinctus*	S1a	E. Bagagli	48
Pb66		Brazil	PCM	S1a	C. de Almeida Soares	49
PbBer	Bercelli; A3	Argentina	PCM	S1a	R. Negroni	5
D02		Laranjal Paulista, SP, Brazil	PCM	S1a	E. Bagagli	This study
T1F1	B1	Pratanea, SP, Brazil	*D. novemcinctus*	S1a	E. Bagagli	48
T15N1		Botucatu, Brazil	*D. novemcinctus*	S1a	E. Bagagli	This study
T16B1		Brazil	*D. novemcinctus*	S1a	E. Bagagli	This study
Pb300^a^	V1	Miranda, Venezuela	Soil	PS4	M. B. Albornoz	50
EPM83		Bogotá, Colombia	Chronic PCM	PS3	A. Restrepo	49
Pb339	B18	Sao Paulo, Brazil	PCM	PS3	A. Restrepo	51
Pb60855	C4	Antioquia, Colombia	Chronic PCM	PS3	A. Restrepo	52
PbBac		Colombia	PCM	PS3	A. Restrepo	This study
PbCab	P196; C6	Caldas, Colombia	*C. centralis*	PS3	A. Restrepo	53
PbCnh^a^		Colombia	Chronic PCM	PS3	A. Restrepo	This study
PbJam		Colombia	Chronic PCM	PS3	A. Restrepo	This study
Pb02	V2	Caracas, Venezuela	Chronic PCM	PS2	R. Puccia	54
Pb03^a^	B26	Sao Paulo, Brazil	Chronic PCM	PS2	R. Puccia	54
Pb262		Uberlândia, MG, Brazil	Dog food	PS2	Z. Pires de Camargo	55
T10B1	B7	Botucatu, Brazil	*D. novemcinctus*	PS2	E. Bagagli	5
*P. lutzii*						
Pb01^a^		Goiás, Brazil	PCM	*P. lutzii*	R. Puccia	56
Pl1578		Goiás, Brazil	PCM	*P. lutzii*	C. de Almeida Soares	47
ED01		Goiás, Brazil	PCM	*P. lutzii*	C. de Almeida Soares	47
PlEE	EE	Mato Grosso, Brazil	PCM	*P. lutzii*	M. Sueli Felipe	4

a Reference strain assembled and annotated genome.

b PCM, paracoccidioidomycosis.

To better understand the evolutionary history of *Paracoccidioides*, we estimated the divergence and most common ancestor dates (time to most recent common ancestor [TMRCA]). This analysis suggests that *P. lutzii* and *P. brasiliensis* diverged about 22.5 million years ago (MYA). The *P. brasiliensis* lineages separated more recently at 1.47 MYA (Fig. 1). Within *P. brasiliensis* lineages, the shortest time of divergence was found for PS3 (TMRCA, 23,000 years ago [23 KYA]) and the longest was found in S1b (TMRCA, 575 KYA), with PS2 and S1a showing intermediate values (Fig. 1; see Fig. S4 in the supplemental material). We estimated that *P. lutzii* diverged slightly earlier than the *P. brasiliensis* groups, around 833 KYA. Using whole-genome SNPs, the separation of *P. brasiliensis* and *P. lutzii* is very similar to a previously reported estimate (8); however, we estimate more recent separation within each lineage.

10.1128/mSphere.00213-16.6 Figure S4  Divergence and most common ancestor dates in *Paracoccidioides*. Bayesian phylogenetic trees were used to estimate the time to most recent common ancestor (TMRCA) for *P. brasiliensis*, *P. lutzii*, and *P. brasiliensis* lineages S1a, S1b, PS2, and PS3. Download Figure S4, PDF file, 1.1 MB. Copyright © 2016 Muñoz et al. 2016 Muñoz et al. This content is distributed under the terms of the Creative Commons Attribution 4.0 International license.

While the SNP data strongly supported a single tree, we also examined the relationship of the sequenced isolates using a network approach to look for evidence of alternative topologies. The NeighborNet algorithm identified five major groups within the *P. brasiliensis* species, including the clear separation of S1 into the S1a and S1b lineages. In addition, the network suggests some level of ancestral recombination between the groups as well as more recent recombination involving the S1b lineage (Fig. 1B). We tested for evidence of phylogenetic heterogeneity using the pairwise homoplasy index test (15) and found statistically significant support for recombination (*P* = 4.3e−13).

**FIG 2:**
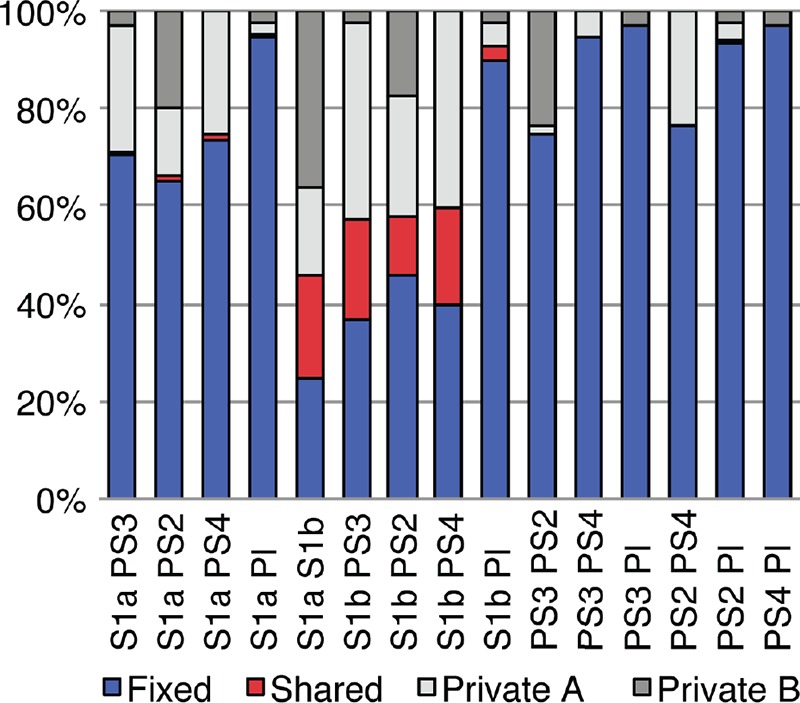
Classification of population-specific SNP alleles. The percentage of the distribution of SNP alleles is shown for each pairwise comparison among the six lineages of *Paracoccidioides* spp. SNP alleles were classified as fixed (blue), shared (red), private A (light gray [for each pairwise comparison specific to the first lineage in the *x* axis]), and private B (dark gray [specific to the second lineage]).

Next, we compared the distribution of SNPs across the genome and examined how sites were shared between lineages. We found a similar pattern of polymorphism frequency across the genome for isolates of the same lineage; differences between lineages include two distinct patterns for the related phylogenetic lineages S1a and S1b, supporting this subdivision (see Fig. S2 in the supplemental material). To more finely compare variant sites between lineages, we classified SNP alleles based on populations as fixed, shared, or private based on pairwise comparisons between lineages (S1a, S1b, PS2, PS3, PS4, and *P. lutzii*). S1b has the highest shared SNP allele component, ranging from 12.4% to 21.1% compared with the S1a, PS2, PS3 and PS4 lineages and 2.5% compared with *P. lutzii* (Fig. 2). In contrast, the next highest shared frequency is for S1a, which ranged from 0.17% to 1.1% compared with PS2, PS3, PS4, and *P. lutzii*. To determine whether the large fraction of shared alleles was due to recombination rather than just a higher genetic diversity in S1b, we combined SNPs for PS3 and PS4 to make an artificial lineage with diversity relatively equivalent to that of S1b. In this combined set, we did not observe the fraction of shared alleles increase from the values of PS3 and PS4 alone, suggesting the effect in S1b is not due to high diversity alone. While recombination with other lineages is also supported by the network tree analysis, it is unclear whether this recombination was relatively recent or ancestral.

### Recombination and hybridization between the major lineages of *Paracoccidioides.*

**FIG 3:**
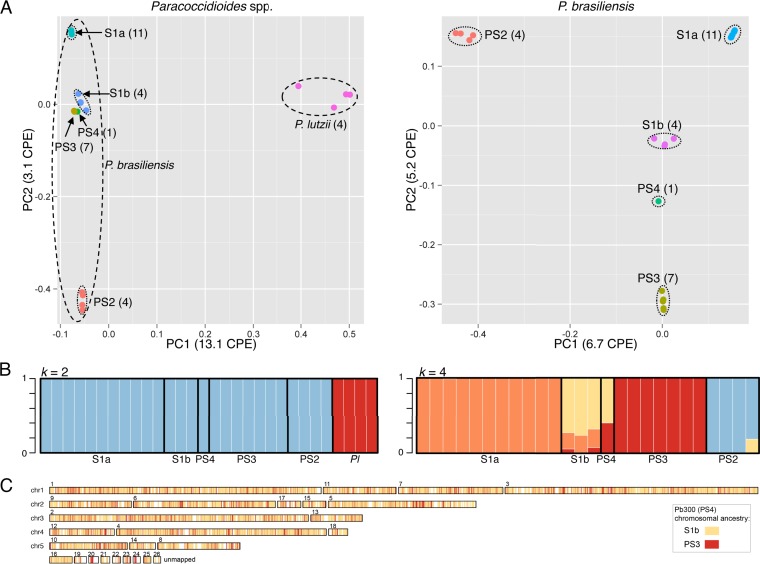
Genetic population structure of *Paracoccidioides*. (A) PCA of genetic variants in *Paracoccidioides* is shown as a two-dimensional plot for all isolates (*n* = 31) (left) or for all *P. brasiliensis* isolates (*n* = 26) (right). In each plot, circles indicate isolates and colors indicate the lineage. (B) Population structure of *Paracoccidioides* spp. (left) and *P. brasiliensis* (right) inferred from 476,589 and 339,966 SNPs, respectively, using the Structure software program with different *k* values of 2 and 4, respectively. An admixture model with correlated allele frequencies and site-by-site analysis was used. Each isolate is represented by a single vertical line broken into *k*-colored segments, with lengths proportional to each of the *k* inferred clusters. (C) Whole-genome plot for Pb300 (PS4) deducted from Structure site-by-site analysis showing the chromosomal ancestry.

To further examine the *Paracoccidioides* lineages for evidence of recombination, we performed a principal-component analysis (PCA) of the SNP data. For all *Paracoccidioides* isolates (*n* = 31), we found clear separation of the two species; PC1 cleanly separates *P. brasiliensis* and *P. lutzii* isolates as two distinct species. Within *P. brasiliensis*, PC2 separates PS2 from all other groups and divides S1 into two distinct groups (S1a and S1b lineages) (Fig. 3A). In this comparison, the S1b, PS3, and PS4 lineages appeared more closely related, suggesting less genetic divergence between these lineages. Comparing only the *P. brasiliensis* isolates (*n* = 26), PC2 separates the S1b, PS4, and PS3 lineages, where PS4 is located between S1b and PS3 (Fig. 3A). This analysis supports the major subdivisions in the *P. brasiliensis* population and suggests that some lineages have similar relationships to multiple other lineages, appearing more centrally in PCA plots, suggesting the impact of recombination.

To examine the relationships of these groups, we predicted the population ancestry for each strain using a model-based clustering algorithm implemented by the Structure software (16). We identified populations within *Paracoccidioides* spp. using (i) all isolates and (ii) only *P. brasiliensis* isolates. There is clear separation between *P. brasiliensis* and *P. lutzii*, with only two major clusters of ancestry (Fig. 3B). When only *P. brasiliensis* isolates are examined, four distinct ancestry clusters are most highly supported (see Materials and Methods), corresponding to S1a, PS2, PS3, and partially S1b. Evaluation of different values of *k* showed overall support for these major subdivisions; however, *P. brasiliensis* could also be inferred to have 3 primary clusters of S1a, PS2, and PS3 (see Materials and Methods and Fig. S5 in the supplemental material). In both the 3- and 4-cluster analyses, isolates from S1b and PS4 have a subset of sites found in different clusters; there is a unique set of S1b/PS4 sites in the 4-cluster analysis, similar to the private alleles found for S1b (Fig. 2). These patterns support a hybrid ancestry for the S1b and PS4 *P. brasiliensis* isolates, which share SNP markers in different proportions with the S1a and PS3 groups (Fig. 3B). Plotting SNPs colored by ancestry across the genome for the PS4 isolate Pb300 revealed a highly intermixed pattern of small blocks of S1b and PS3 ancestry, suggesting a relatively ancient hybridization event (Fig. 3C).

10.1128/mSphere.00213-16.7 Figure S5  *Paracoccidioides* population genetic structure for a range of fixed cluster (*k*) sizes. (A) Cluster representation of *Paracoccidioides* spp. (left) and *P. brasiliensis* (right) inferred from 476,589 and 339,966 SNPs, respectively, using the program structure and plotted with MavericK, with different *k* values ranging from 2 to 5. (B) Thermodynamic integration estimates using MavericK of the model evidence in log space (left) and in linear space (right) after normalizing to sum to 1 using multiple replicates of 1, 2, and 10% subsamples of the 339,966-SNP *P. brasiliensis* matrix. Download Figure S5, PDF file, 0.2 MB. Copyright © 2016 Muñoz et al. 2016 Muñoz et al. This content is distributed under the terms of the Creative Commons Attribution 4.0 International license.

### Equal frequencies of mating types in each lineage.

Although the sexual phase of *Paracoccidioides* had not been completely characterized in nature or in the laboratory, the patterns of recombination and ancestral hybridization we found may be most parsimoniously explained by sexual reproduction. We identified the mating type of each isolate based on the genomic sequenced data. While the previously sequenced reference genomes Pb18 (S1b) and Pb03 (PS2) contain the mating type HMG (*MAT1-2*), here we generated the first assembled genome of mating type α (*MAT1-1*) for *P. brasiliensis* (strain PbCnh [PS3]). By aligning the assemblies, we observed that there are not any chromosomal rearrangements near the mating locus that could impact the capacity for interlineage genetic exchange (see Fig. S1F in the supplemental material). The assembly of Pb300 (PS4) also contains the mating type HMG (*MAT1-2*). As previously noted, conservation of mating- and meiosis-specific genes suggests that *P. brasiliensis* has the necessary machinery for sexual reproduction (12), and we see no additional gene loss in these lineages. All of the 31 sequenced *Paracoccidioides* isolates were heterothallic with a population ratio of 1:1 of each mating type, both in the population as a whole as well as among each lineage, with a total of 15 isolates α (*MAT1-1*) and 16 isolates HMG (*MAT1-2*) (see Fig. S6 in the supplemental material). The evidence for genetic exchange and recombination and these roughly equal numbers of both mating types in each lineage support the potential for sexual reproduction in *Paracoccidioides*.

10.1128/mSphere.00213-16.8 Figure S6  Mating-type locus for sexual reproduction. The numbers of mapped positions for all sequenced *Paracoccidioides* isolates mapped to each of the PbCnh and Pb18 reference strains were computed for each specific locus from VCF files, α *MAT1-1* (GX48_02691), and HMG box *MAT1-2* (PADG_06118), respectively. Download Figure S6, PDF file, 0.3 MB. Copyright © 2016 Muñoz et al. 2016 Muñoz et al. This content is distributed under the terms of the Creative Commons Attribution 4.0 International license.

### Genome-wide population genetic variation between the major lineages of *Paracoccidioides.*

**FIG 4:**
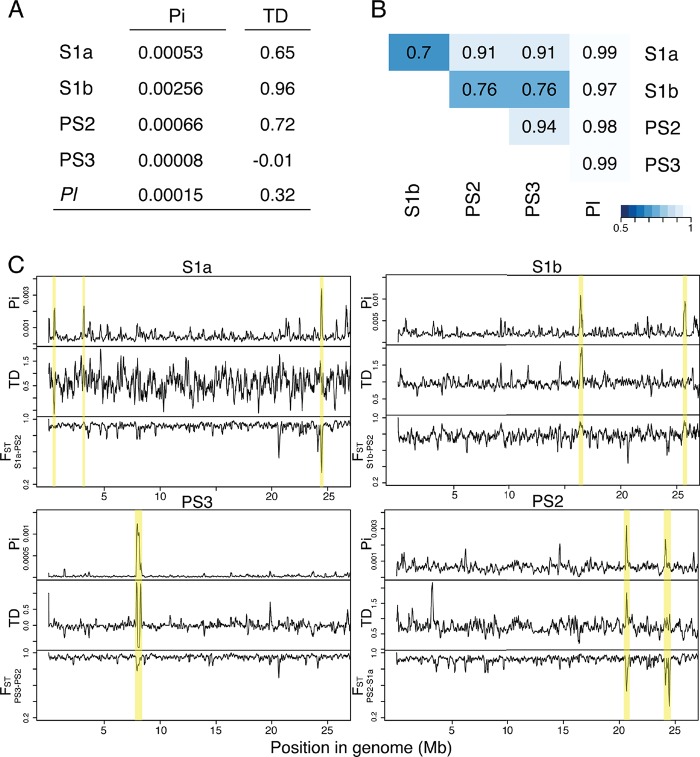
Genome-wide nucleotide diversity (π [Pi]), Tajima’s *D* (TD), and population divergence analysis (*F_ST_*) in *Paracoccidioides*. (A) Genome-wide average of nucleotide diversity (π) and TD for sliding windows of 10-kb regions within the main *Paracoccidioides* lineages. (B) Average of genome-wide (10-kb windows) variation in (θ), Weir’s formulation of Wright’s fixation index (*F_ST_*), for pairwise comparisons in each lineage. (C) Distribution of the average nucleotide diversity (π), TD, and *F_ST_* for sliding windows of 10-kb regions for the S1a, S1b, PS3, and PS3 *Paracoccidioides* lineages.

Both polymorphism and phylogenetic analyses suggest that PS3 is a monophyletic group derived from a shared common ancestor with limited diversity, and S1b is the most variable lineage, reflecting a more widespread geographic distribution. We found the highest level of nucleotide diversity in S1b (π = 0.00256), which was 30-fold greater than the very low nucleotide diversity found in PS3 (π = 0.00008). Nucleotide diversities in S1a, PS2, and *P. lutzii* were intermediate: 0.00053, 0.00066, and 0.00015, respectively (Fig. 4A). In addition, we tested for genome-wide allele frequency distribution using Tajima’s *D* (TD) to scan for signatures of demography and selection. We found that all populations showed genome-wide Tajima’s *D* values not significantly different from the null expectation. This suggests that each lineage is evolving neutrally at the whole-genome level (Fig. 4A).

Additionally, we analyzed interspecific divergence between *P. brasiliensis* and *P. lutzii* and between *P. brasiliensis* lineages (S1a, S1b, PS2, and PS3) across the genome using Wright’s *F_ST_* statistic and a 10-kb sliding-window approach. Across the *P. brasiliensis* pairwise comparisons, values ranged between 0.70 and 0.94, suggesting limited genetic exchange between the lineages (Fig. 4C; see Fig. S7 in the supplemental material). Furthermore, there were no regions of very low *F_ST_* that would indicate recent genetic exchange (see Fig. S7). Comparisons of *P. lutzii* and *P. brasiliensis* lineages had values ranging from 0.97 to 0.99, suggesting strong local divergence between these species, similar to the 0.95 *F_ST_* value observed for most regions of *Coccidioides immitis* compared to *Coccidioides posadasii* (17). S1b had the lowest pairwise *F_ST_* values (0.70 to 0.76) compared to the remaining lineages (0.91 to 0.94) (Fig. 4B; see Fig. S7). This supports the hypothesis of recombination between S1b and the other lineages, which correlates with the intermediate position of S1b in the population structure analysis and higher degree of shared alleles (Fig. 3 and 2, respectively). This may have clinical implications since the S1b lineage is widely distributed in South America, includes highly virulent strains, and has been associated with the vast majority of cases of PCM (Fig. 1).

10.1128/mSphere.00213-16.9 Figure S7  Genome-wide population divergence analysis (*F_ST_*) in *Paracoccidioides*. Shown is the *F_ST_* calculated for pairwise group comparisons with reference to the Pb18 chromosome anchored assembly. Download Figure S7, PDF file, 0.5 MB. Copyright © 2016 Muñoz et al. 2016 Muñoz et al. This content is distributed under the terms of the Creative Commons Attribution 4.0 International license.

### Lineage-specific gain and loss and rapid evolution of virulence-associated genes.

Comparing the *P. brasiliensis* lineages, a total of 6,670 core ortholog clusters had representative genes from all five reference genomes. We found 720 ortholog groups in at least two strains and 459 ortholog groups that were present in all *P. brasiliensis* strains but absent in *P. lutzii*. We did not find any significant enrichment of functional categories among *P. lutzii* and *P. brasiliensis* strains or among their lineages, which suggests that the phenotypic differences between *P. lutzii* and *P. brasiliensis* and between the lineages are not due to large protein family expansion or contraction. However, unique genes (those without orthologs in other lineages) could contribute to different phenotypic differences observed in each lineage, as well as genes duplicated in only one species. Among the unique genes we identified, there were genes coding for several protein kinases, proteases, transcription factors, and transporters (see Data Set S1 in the supplemental material). More specifically, in *P. lutzii* there were several unique genes coding for anhydrolases, glycosyl hydrolases, peptidases (M24 and C12), and methyltransferases, while in *P. brasiliensis* there were several unique genes coding for actin, transporters, aspartyl proteases, peptidases (M16 and M28), and transcription factors. These unique proteins may provide a more diverse functional repertoire to each *P. lutzii* or *P. brasiliensis* lineage, enabling the fungus different mechanisms and strategies to produce infection and disease.

In addition to identifying strain-specific genes, we identified nonsense mutations that were specific to each lineage. For example, the serine carboxypeptidase gene (*CPDS;* PADG_07980), the predicted mannan endo-1,6-α-mannosidase gene (PADG_00193), and the predicted transmembrane gene (PADG_08161) have nonsense mutations in all lineages of *P. brasiliensis* (S1a, S1b, PS2, PS3, and PS4) but not in *P. lutzii*, suggesting these genes are not functional in *P. brasiliensis*. Other genes that do not have nonsense mutations in *P. brasiliensis* (S1a, S1b, PS2, PS3, and PS4) but have nonsense mutations in all *P. lutzii* strains included genes coding for the separase protease (peptidase_C50; PADG_07698), a DNA-binding protein protease (peptidase_M24; PADG_01032), other secreted proteins (e.g., PADG_00436), as well as other genes coding for proteins involved in different cellular processes (e.g., sugar transporter, PADG_04202; sterigmatocystin biosynthesis monooxygenase StcW, PADG_04703; and a glucose-6-phosphate isomerase, PADG_00451) (see Data Set S1). Differences in gene content between the species, including genes coding for predicted proteases and cell wall genes, could impact how each species/lineage interacts with the host or environment, although other genes with overlapping functions could compensate for loss of these genes.

**FIG 5:**
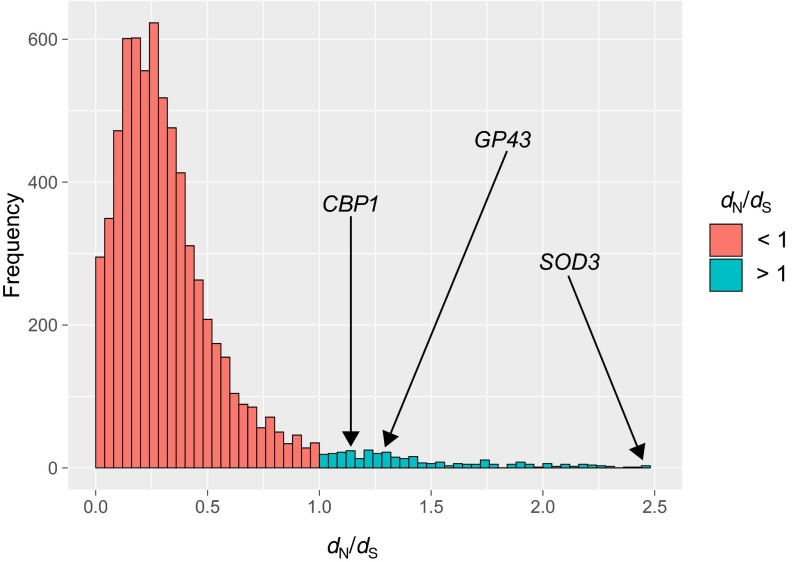
Genes under positive selection in *Paracoccidioides*. Shown is a histogram of the *dN*/*dS* values comparing *P. brasiliensis* (Pb18) and *P. lutzii* (Pb01). Genes undergoing positive selection (*dN*/*dS* of >1) are in blue.

To more finely examine gene sequence differences and the impact of selection, we calculated the ratio of nonsynonymous to synonymous evolutionary changes (*dN*/*dS*_)_ for each gene across the lineages of *Paracoccidioides*. An average of 485 genes were found to be under positive selection (*dN*/*dS* of >1) in each of the lineages (Fig. 5; see Data Set S1). The set of genes evolving under positive selection includes the surface antigen gene *GP43* (PADG_07615), the superoxide dismutase gene *SOD3* (PADG_02842), the alternative oxidase gene *AOX* (PADG_03747), and the thioredoxin gene (PADG_05504), virulence-associated genes of central importance in *Paracoccidioides* and other dimorphic fungi (Table 2). Other notable genes under positive selection included several protease genes, including a glutamate carboxypeptidase gene (PADG_00686), a multifunctional tryptophan biosynthesis protein gene (PADG_07274), the a-pheromone processing metallopeptidase gene *STE23* (PADG_07053), and the subtilase-type proteinase gene *PSP3* (PADG_07422). In addition, many genes coding for secreted proteins appear under positive selection, including the calcium binding protein gene *CBP1* (PADG_02399) and the mannan endo-1,6-α-mannosidase gene *DCW1* (PADG_01494). Among the 88 total secreted proteins under positive selection, seven were found to be significantly differentially upregulated during a mouse model of infection in *Blastomyces* (14), including a high-affinity nickel transporter (PADG_05345), an oxidoreductase (PADG_00948) and the *SOD3*-encoded protein. Other genes that were found significantly induced during the interaction of *Blastomyces* with macrophages (14) were also found to evolve under positive selection in *Paracoccidioides*, including the two most highly upregulated in infected macrophages, a gene coding for a secreted protein with unknown function (PADG_01283) and a secreted endo-1,3(4)-β-glucanase gene, *ENG1* (PADG_12370); other genes found in both analyses include genes coding for GATA-binding protein (PADG_05497), an aldehyde reductase (PADG_01835), and a glucan 1,3-β-glucosidase (PADG_06699) (Table 2; see Data Set S1). These genes therefore may be generally important for host interactions of dimorphic pathogens and make good candidates for future studies on their contribution to virulence.

**TABLE 2:** Selection of candidate virulence factors in *Paracoccidioides* isolates found in highly diverse regions and/or under selection

Locus ID	Description of protein	π	TD	SNPs	*dN*/*dS* >1	*F_ST_*
PADG_02498	3-Hydroxyanthranilate 3,4-dioxygenase	+	−	−	−	−
PADG_00940	Acetate kinase; *B. dermatitidis* ortholog induced during macrophage interaction	−	+	−	+	−
PADG_01835	Aldehyde reductase; *B. dermatitidis* ortholog induced during macrophage interaction	−	−	−	+	+
PADG_07461	α-1,3-Glucanase	+	+	+	+	+
PADG_03747	Alternative oxidase; *AOX* gene	−	−	−	+	−
PADG_02460	Antigenic GPI-protein; secreted; antigen; *PGA1* gene	+	−	+	+	+
PADG_04167	Aspartyl aminopeptidase; peptidase family M18	+	−	−	−	−
PADG_06131	BUD32 protein kinase; vesicles	−	−	−	+	−
PADG_02399	Calcium binding protein; secreted; *CBP1 gene*	−	−	−	+	+
PADG_00743	Class II aldolase; *B. dermatitidis* ortholog induced during macrophage interaction	−	−	−	+	−
PADG_12370	Endo-1,3(4)-β-glucanase; secreted; *ENG1* gene; *B. dermatitidis* ortholog induced during macrophage interaction	−	+	−	+	−
PADG_05497	GATA-binding protein; *B. dermatitidis* ortholog induced during macrophage interaction	−	−	−	+	−
PADG_07615	Glucan 1,3-β-glucosidase; secreted; antigen; *GP43* gene; induced during macrophage interaction	−	+	+	+	−
PADG_05345	High-affinity nickel transporter; *B. dermatitidis* ortholog induced during *in vivo* infection	−	−	−	+	−
PADG_07274	Hypothetical protein	−	−	−	+	−
PADG_06699	Hypothetical protein; *B. dermatitidis* ortholog induced during macrophage interaction	−	+	−	+	−
PADG_01238	Hypothetical protein; *B. dermatitidis* ortholog induced during macrophage interaction	−	−	−	+	−
PADG_01283	Hypothetical protein; *B. dermatitidis* ortholog induced during macrophage interaction	−	−	−	+	−
PADG_03908	Hypothetical protein; *B. dermatitidis* ortholog induced during macrophage interaction	−	−	−	+	−
PADG_07534	Hypothetical protein; *B. dermatitidis* ortholog induced during macrophage interaction	−	−	−	+	−
PADG_11963	Hypothetical protein; *B. dermatitidis* ortholog induced during macrophage interaction	−	−	−	+	+
PADG_02535	Hypothetical protein; secreted; vesicles; *B. dermatitidis* ortholog induced during *in vivo* infection	+	+	+	+	+
PADG_02542	Hypothetical protein; vesicles	−	−	−	+	+
PADG_12450	Hypothetical protein; vesicles	+	−	+	+	−
PADG_04741	Hypothetical protein; vesicles	+	+	−	+	−
PADG_02521	Hypothetical protein; vesicles	+	−	−	+	−
PADG_12101	Hypothetical protein; vesicles	−	−	−	+	−
PADG_01494	Mannan endo-1,6-α-mannosidase; secreted; *DCW1* gene	−	+	−	+	−
PADG_00948	Oxidoreductase; *B. dermatitidis* ortholog induced during *in vivo* infection	−	+	−	+	−
PADG_07460	Predicted aminopeptidase; peptidase family M18; induced during macrophage interaction	+	+	+	−	+
PADG_05820	Predicted aminopeptidase; peptidase family M24; induced during macrophage interaction	+	−	+	−	−
PADG_07369	Predicted dehydrogenase	+	+	+	−	+
PADG_02527	Predicted dehydrogenase	+	−	−	−	−
PADG_02562	Predicted dehydrogenase	+	+	−	−	−
PADG_02492	Predicted dehydrogenase	+	−	−	−	−
PADG_07365	Predicted dehydrogenase	+	−	−	−	+
PADG_07411	Predicted dehydrogenase	+	−	−	−	−
PADG_02575	Predicted nonribosomal peptide synthetase	+	+	−	−	−
PADG_06309	Predicted oxidoreductase	+	+	+	−	+
PADG_02592	Predicted oxidoreductase	+	+	+	−	−
PADG_06322	Predicted peroxidase	+	+	−	−	+
PADG_02507	Predicted peroxidase; secreted	+	−	−	+	−
PADG_07053	Predicted protease; peptidase family 16	−	−	−	+	−
PADG_00686	Predicted protease; peptidase family M28	−	−	−	+	+
PADG_06314	Predicted protease; peptidase family S10; secreted; induced during macrophage interaction	+	+	−	−	−
PADG_06167	Predicted protease; peptidase family S24	+	−	−	+	−
PADG_07422	Predicted protease; peptidase family S8; secreted	−	+	−	+	+
PADG_07454	Predicted scramblase; secreted	+	−	+	+	+
PADG_06308	Predicted transporter	+	+	+	+	+
PADG_02081	RING finger domain-containing protein; vesicles	−	+	−	+	−
PADG_08583	Secreted protein	−	−	−	+	−
PADG_02569	Secreted protein	+	+	−	−	−
PADG_00954	Secreted protein immunoreactive protein; secreted	+	+	+	+	−
PADG_07830	Secreted protein; *B. dermatitidis* ortholog induced during *in vivo* infection	−	−	−	+	−
PADG_05055	Secreted protein; *B. dermatitidis* ortholog induced during macrophage interaction	+	+	+	+	−
PADG_03277	Secreted protein; vesicles; *B. dermatitidis* ortholog induced during *in vivo* infection	−	−	−	+	−
PADG_02842	Superoxide dismutase; secreted; *SOD3* gene; *B. dermatitidis* ortholog induced during *in vivo* infection	−	−	−	+	−
PADG_05504	Thioredoxin	−	−	−	+	−
PADG_02515	Transporter	+	−	−	+	−
PADG_05881	Vacuolar amino acid transporter; vesicles	−	−	−	+	−
PADG_00941	Xylulose-5-phosphate phosphoketolase; *B. dermatitidis* ortholog induced during macrophage interaction	−	+	−	+	−

### Combining diversity measures reveals lineage-specific targets of positive selection.

Examination of the local variation in nucleotide diversity (π) and Tajima’s *D* (TD) across the genome revealed small regions with high lineage-specific diversity, which also typically showed high TD (Fig. 4C; see Data Set S1 in the supplemental material). PS2 had the most lineage-specific high-diversity windows, with 14 in total, coinciding with the more ancient divergence of this lineage and in agreement with the phylogenetic analysis. The windows with significantly high π and TD highlighted lineage-specific regions of high diversity and selection, which in some cases were regions with low interspecific divergence (*F_ST_*) (Fig. 4B).

These high-diversity regions highlighted different sets of variable and rapidly evolving genes within lineage. These genes encompass diverse cellular functions, including coding for secreted and cell wall proteins, transport, transcription regulation, oxidative stress, and proteolysis (Table 2; see Data Set S1), some of which have experimental evidence of a role in virulence and pathogenicity (see Text S1 in the supplemental material). Two high-diversity regions found for S1b include two aminopeptidases (PADG_05820 and PADG_07460) previously noted to be upregulated in *Paracoccidioides* during macrophage infection (18). In the low-diversity PS3 isolates, one large highly variable region was identified that includes 125 genes (23 with a *dN/dS* of >1, 12 secreted protein genes, and 9 genes involved with the oxidation reduction process). Among these genes are genes encoding two glycosylphosphatidylinositol (GPI)-anchored proteins (including *PGA1*) and the secreted protein PADG_02535, which was identified in extracellular vesicles of *Paracoccidioides* (19), and it was recently shown in *B. dermatitidis* that an ortholog was more highly expressed during mouse pulmonary infection (14). In two regions found for PS2, 11 of 48 genes show evidence of positive selection (*dN*/*dS* of >1) and include genes coding for a serine carboxypeptidase (PADG_06314) induced in *Paracoccidioides* during macrophage infection (18) and a peroxidase (PADG_06322), as well as the amino acid permease PADG_07440, which in *B. dermatitidis* was highly induced during mouse pulmonary infection (14). Differences in these genes might impact the virulence phenotypes observed across the lineages and make good candidates for future studies of their contribution to virulence variation between isolates of *Paracoccidioides* (Table 2).

## DISCUSSION

Building on previous genomic analysis of *Paracoccidioides* (12, 13), here we reevaluate the major lineages and provide new reference genomes for two lineages, PS3 and PS4. Together these data enable a more comprehensive view of the genome content of *Paracoccidioides* and help trace the genome-level variation following the recent divergence into well-defined lineages. We find clear support for the separation of *P. brasiliensis* into distinct lineages; however, we also find evidence of recombination and highest diversity within a single lineage, the newly described S1b. Dating the timing of the separation of each lineage supported S1b as the earliest diverging *P. brasiliensis* lineage, with the later emergence of PS2, S1a, and PS3.

Our study provides additional support for *P. brasiliensis* and *P. lutzii* as separate species, with some amount of incomplete lineage sorting suggested by intermediate *F_ST_* values between the *P. brasiliensis* lineages. Species definitions have been reevaluated in many fungal groups, including the *Onygenales* (20), based on improved phylogenies from wider sets of isolates compared or incorporation of additional loci, or even whole genomes, in addition to the analysis of morphological data of sexual and asexual structures and the evidence of sexual reproduction. Using multiple approaches, we see clear separation of *P. brasiliensis* and *P. lutzii* and evidence of recombination within *P. brasiliensis*; however, this recombination did not appear to be recent. While the phylogenetic separation suggests the *P. brasiliensis* lineages are largely genetically isolated, increased sample size is required to compare variation within and between the lineages to support their separation into different species.

We found support for a split in the S1 group into S1a and S1b lineages; in addition to genetic separation, signatures of recombination clearly differentiate S1b from S1a. While the phylogenetic analysis suggests that S1a and S1b are both monophyletic, we also find evidence from multiple analyses that S1b, the most geographically widespread lineage, has undergone recombination with each of the other lineages. This appears to be mostly ancestral, as *F_ST_* while variable did not reveal any large recently introgressed regions between lineages. The mixed ancestry of PS4 also suggests that more ancient recombination created this lineage, as the pattern of SNP ancestry across the genome revealed an intermixed distribution of both S1b and PS3 alleles that were likely shuffled by extensive recombination over time. The sequence of additional isolates of PS4 as well as S1b could help further explore how alleles within these groups are distributed within each lineage and across geographic regions.

All lineages, including S1a and S1b, contain roughly equal numbers of both mating types based on the sequenced isolates; an equal ratio was also previously noted in a wider set of *P. brasiliensis* isolates (21). The equal representation suggests that other differences between the strains explain how S1b has undergone higher levels of recombination compared to S1a. Direct testing of mating potential for different strains requires the further development of laboratory mating experiments, which appear to only initiate but not complete mating to date (22). In addition, further studies comparing larger numbers of isolates from the same geographic region but different lineage groups would be more sensitive to detecting recent exchange between groups in nature.

This genome-wide comparison of the major lineages of *Paracoccidioides* revealed that while the genome organization and gene content are highly conserved, specific genes under positive selection include several previously known to be important for host interaction as well as new candidates. Two of the genes we identified (*GP43* and *CTS20*) were noted in a previous study, which utilized expressed sequence tags (ESTs) and found evidence of positive selection in 11 of 32 examined genes implicated in virulence (23). Here, we find evidence for positive selection in an average of 485 genes per strain using the updated genome sequences. In addition to *GP43*, we see evidence of selection in the gene coding for the secreted antigenic GPI-anchored protein, *PGA1* (24). Other genes under positive selection include those coding for aminopeptidases highly upregulated during host-pathogen interactions (14), secreted proteins previously found in *Paracoccidioides* extracellular vesicles (19), and other proteins involved in mitigating oxidative stress that were also found induced during the interaction with macrophages, such as the *AOX*, *SOD3*, and *CBP1* genes (14, 18, 25–27). Secreted proteins in *Paracoccidioides* have been associated with nutrient acquisition, cell defense, and modulation of the host defense machinery.

Better knowledge of the differences between the *Paracoccidioides* lineages can help guide the development of new diagnostics, treatments, and models of pathogen evolution. Delineation of the prevalent S1 group into the well-separated S1a and S1b lineages allowed us to differentiate S1b as the more highly recombining and diverse lineage. The higher diversity present in S1b may contribute to its dispersion and survival in a wider geographical range than the other lineages, perhaps enabling local adaptation such as to temperature variation. In our data, all of the S1b strains are clinical isolates and include two highly virulent strains, Pb18 and Pb113, and two strains from acute PCM cases (PbBlo and PbCaz). In contrast, the S1a lineage includes clinical and armadillo isolates, with very few sequence differences between strains from these sources. Sequence of isolates from a wider geographical range, including Brazil, Argentina, Paraguay, and Mexico, would likely increase the sampled genetic diversity of *Paracoccidioides* and allow further study of these trends; additional isolates could also enable genome-wide association studies of clinically relevant phenotypes. Development of sequence-based diagnostics will need to take into consideration that S1b strains share a higher percentage of alleles with all other strains; as these sites could contribute to misidentification of lineages, sufficient power or selection of sites unique to each group that also take into account the higher diversity in S1b would improve diagnostic power.

## MATERIALS AND METHODS

### Selection and sequencing of *Paracoccidioides* isolates.

A total of 31 isolates of *P. brasiliensis* and *P. lutzii* were sequenced and included in the analyses (Table 1): 11 isolates from lineage S1a, 4 isolates from S1b, 7 isolates from PS3, 4 isolates from PS2, 1 isolate from PS4, and 4 isolates from *P. lutzii*. Selected isolates included clinical isolates from acute and chronic PCM cases, environmental isolates from armadillos, and the previously published and updated reference genomes of *P. brasiliensis* (Pb03 from PS2 and Pb18 from S1) and Pb01 from *P. lutzii* (12, 13). Genomic DNA for sequencing was prepared from yeast culture, using phenol-chloroform extraction method. The genomes were sequenced using Illumina sequencing platforms and using different insert sizes (~620, ~180, or ~650 bp), paired-end-read lengths (150, 101, or 100 bp) and sequence coverage ranging from 68 to 419 (see Table S1 in the supplemental material).

10.1128/mSphere.00213-16.10 Table S1  Whole-genome sequencing details for selected *Paracoccidioides* isolates. Download Table S1, XLSX file, 0.1 MB. Copyright © 2016 Muñoz et al. 2016 Muñoz et al. This content is distributed under the terms of the Creative Commons Attribution 4.0 International license.

### Identification and analysis of gene orthologs and selection analysis.

For a detailed description of the genome assembly and gene annotation of *P. brasiliensis* Pb300 and PbCnh strains, see the supplemental material. The five assembled and annotated genomes representing each lineage of the *Paracoccidioides* species were used for comparative analysis. Genes were functionally annotated by assigning Pfam domains, GO terms, KEGG classification, SignalP, and TMHMM. OrthoMCL (28) was used to cluster the protein-coding genes of the four chosen genomes by similarity and create sets of genes that have a high probability of being orthologous to each other.

For selection analysis, variant call format (VCF) files were used to align the reference transcript set in coding triplets in the PHYLIP multiple sequence alignment format. Then we calculated the ratio of nonsynonymous to synonymous substitutions (*dN*/*dS*) within the *Paracoccidioides* genus on properly aligned genes. We employed the yn00 program in PAML (29), implementing the Yang and Nielsen method (30). To eliminate the possibility that fast-evolving genes detected in this analysis were biased for false-positive SNP calls, we calculated the mean base quality, mean mapping quality, and quality normalized to depth and compared these parameters for all fast-evolving genes and all other genes.

### SNP variant detection and analysis.

To detect polymorphisms, we used reference assemblies representing each lineage (Pb18, Pb03, PbCnh, Pb300, and Pb01). Each of the 31 Illumina data sets was independently aligned to the genome assemblies using the short read component *aln* of BWA version 0.5.9 (31) with default settings. SNPs and indels were called with Pilon version 1.4 using the haploid ploidy default setting (32). An average of 38 million reads was aligned per strain, with an average quality and error rate of 33.2 and 2.0E−02, respectively (see Table S1 in the supplemental material). Variant call format (VCF) files were filtered using VCFtools version 0.1.12 (33) or according to further analyses—e.g., considering SNP calls in all strains with genotype 1/1 and minimum depth 4. For SNP positions, the total mapping depth was 125 and the mean base quality was 34, averaged across all variants (see Table S1).

To address if any lineage could be uniformly diploid, we examined candidate heterozygous positions predicted by Pilon. The low frequency of such positions (~0.04%), which often overlap repetitive sequence (68% of these positions), suggests there is little evidence for diploidy. This suggests that all sequenced genomes are homozygous haploids, as expected from prior work establishing that *Paracoccidioides* species are haploid (34, 35).

The false discovery rate (FDR) was estimated as an additional parameter of the mapping and SNP calling accuracy (36). As a truth set, we simulated 670,000 random mutations in the reference genome (Pb18). The number of random mutations was the maximum number of mutations detected with the sequenced strains. Next, we aligned the raw reads to the random-mutated reference, called SNPs, and compared them to the known truth set of simulated mutations to calculate the accuracy of our data and process. Both the number of true positives (658,566; precision, 99.8%, and sensitivity, 98.3%) and the number of false positives (386; 0.06%) show the high accuracy of the overall process, including read quality, alignment accuracy, and the SNP calls.

To determine the chromosomal copy number variation (CCNV) and the distribution of SNPs across the genome, we calculated the alignment density (depth of read coverage) and SNP density (frequency of SNPs per site), respectively. All VCF files were summarized in 10-kb nonoverlapping windows. The alignment density was normalized by the average genomic coverage and the window length. The SNP density was normalized by the window length. The genome assembly of Pb18 is anchored to chromosomes (12); we used this reference to map the alignment density and SNP density into chromosomes. Pb03, PbCnh, Pb300, and Pb01 were mapped into scaffolds.

To more finely compare variant sites between lineages, we classified the distribution of biallelic loci between two populations categorized as “shared,” “fixed,” or “private.” If one allele is present in all members of one population and the other allele is present in all members of the other population, that locus was considered “fixed.” If both alleles are present in both populations, that locus was considered “shared.” If one allele is present in all members of one population and the other population has both alleles, that locus was considered “private.”

To analyze each SNP in the context of gene annotations of the reference genomes we used VCFannotator (http://sourceforge.net/projects/vcfannotator/). Variants were placed in the context of genome feature annotations and indicated as “intergenic,” “intronic,” or “coding.” The type of coding mutation was further characterized by its impact on the protein-coding sequence as “synonymous,” “nonsynonymous,” “nonsense,” or “read-through.” To evaluate the support for each mutation, the mean base quality, the mean mapping quality, and the quality normalized to depth were retrieved and compared.

### Phylogenetic analysis using whole-genome SNPs.

Alignments were constructed from SNP matrices extracted from the VCF format. To consider a position in the alignment matrix, we used a minimum depth of coverage of 4, and we kept those positions with at least one variant site in all the sequenced isolates. We built an SNP alignment matrix for each *Paracoccidioides* reference strain used here representing each lineage to obtain four trees and compare their topologies. Maximum likelihood phylogenies were constructed using RAxML version 8.0.20 (37) using the GTRCAT nucleotide substitution model and bootstrap analysis based on 1,000 replicates. To infer Bayesian phylogenetic trees and to estimate the divergence and most common ancestor dates, we used BEAST v1.8.2 (38). Using the SNP matrices, we normalized the mutation rate for genome-wide variable sites (normalized, 4.43E−8; genome-wide, 1E−9 [39]) that was used as the clock rate along with the coalescent model, relaxed clock/uncorrelated lognormal clock model. For nonpartitioned variant sites, we used the strict clock model, with the constant pattern model, general time reversible (GTR) (G+I) substitution model, and 20,000,000 Markov chain Monte Carlo (MCMC) chains. Tracer v1.6.0 (http://beast.bio.ed.ac.uk/Tracer) was used to visualize traces and inspect the effective sample size and MCMC. We determined the relationship of the sequenced isolates using the NeighborNet algorithm with SplitsTree4 (40).

### Population genetic structure analyses.

We performed a principal-component analysis (PCA) on a matrix of SNP calls for all of the *Paracoccidioides* isolates (*n* = 31) and for only the *P. brasiliensis* isolates (*n* = 26), using SMARTPCA (41). Population structure was performed using the Bayesian model-based clustering program STRUCTURE v2.3 (16) in the site-by-site mode, with successive *k* values from 2 to 6. We identified populations within *Paracoccidioides* using 476,589 SNPs in all isolates and within *P. brasiliensis* isolates using 339,966 SNPs. We estimated the model evidence for *k* using thermodynamic integration method as implemented in MavericK v1.0 (42), using replicates for 10 randomly generated 1% subsamples of the *P. brasiliensis* SNP matrix; this supported *k* = 4 as the best choice for *P. brasiliensis* (see Fig. S5B in the supplemental material). Genome-wide nucleotide diversity (π) and Tajima’s *D* were computed for each identified *Paracoccidioides* population (S1a, S1b, PS2, PS3, and *P. lutzii*) using VCFtools v0.1.12 (33). The average nucleotide diversity (π) and Tajima’s *D* were computed for nonoverlapping sliding windows of 10 kb. We calculated Wright’s fixation index (*F_ST_* [43]) according to the equations given in VCFtools v0.1.12 (33) adjusted for low sample sizes. Sliding-window *F_ST_* analyses were conducted using all SNPs found within 10-kb nonoverlapping windows. Variant call format (VCF) files were filtered using an in-house perl script to calculate *F_ST_*, and clusters for comparison were chosen based on the whole-genome phylogenetic tree.

### Accession number(s).

The assemblies and annotations of the *P. brasiliensis* genomes have been deposited in DDBJ/ENA/GenBank under the following accession numbers: *Paracoccidioides brasiliensis* PbCnh, LYUC00000000; *Paracoccidioides brasiliensis* Pb300, LZYO00000000. All of the whole-genome sequence (WGS) raw data for the 31 *Paracoccidioides* strains have been deposited in the NCBI Sequence Read Archive (BioProject, PRJNA322632; SRA, SRP077566). BioSample and SRA accession numbers for individual strains are included in Table S1 in the supplemental material.
