# Supplementary material for: Genome Diversity, Recombination, and Virulence across the Major Lineages of Paracoccidioides
Source: mSphere. 2016 Sep 28;1(5):e00213-16. doi: 10.1128/mSphere.00213-16 (PMC5040785; doi:10.1128/mSphere.00213-16)
Supplement: Text S1 [file sph005162156s1.docx]

**Supplementary information for Muñoz et al. “Genome diversity, recombination and virulence across the major lineages of *Paracoccidioides*”.**

José F. Muñoz^1,2,3^, Rhys A. Farrer^3^, Christopher A. Desjardins^3^, Juan E. Gallo^1,4^, Sean Sykes^3^, Sharadha Sakthikumar^3^, Elizabeth Misas^1,2^, Emily A. Whiston^5^, Eduardo Bagagli^6^, Celia M. A. Soares^7^, Marcus de M. Teixeira^8,9^, John W. Taylor^5^, Oliver K. Clay^1,10^, Juan G. McEwen^1,11^, Christina A. Cuomo^3,*^

^1^ Cellular and Molecular Biology Unit, Corporación para Investigaciones Biológicas, Medellín, Colombia. ^2^ Institute of Biology, Universidad de Antioquia, Medellín, Colombia. ^3^ Broad Institute of MIT and Harvard, Cambridge, Massachusetts, USA. ^4^ Doctoral Program in Biomedical Sciences, Universidad del Rosario, Bogotá, Colombia. ^5^ Department of Plant and Microbial Biology, University of California, Berkeley, Berkeley, California, United States of America. ^6^ Instituto de Biociências, Universidade Estadual Paulista, Botucatu, SP, Brazil. ^7^ Laboratório de Biología Molecular, Instituto de Ciências Biológicas, ICBII, Goiânia, Brazil. ^8^ Instituto de Ciências Biológicas, Universidade de Brasília, Brasília, DF, Brazil. ^9^ Division of Pathogen Genomics, Translational Genomics Research Institute-North, Flagstaff, Arizona, USA. ^10^ School of Medicine and Health Sciences, Universidad del Rosario, Bogotá, Colombia. ^11^ School of Medicine, Universidad de Antioquia, Medellín, Colombia.

* Corresponding author: cuomo@broadinstitute.org

**Supplementary Notes**

**The genomes of the *P. brasiliensis* PS3 and PS4 lineages**

Genome assembly: All sequenced genomes from isolates of the PS3 and PS4 lineages were assembled *de novo* using SOAPdenovo2 r240 and *k-mer* values ranging from 17 to 49 (1). The genome of Pb300 from PS4 lineage was additionally assembled using SPAdes assembler v 3.1.1 (2) and SSPACE scaffolder with a minimum number of links 10 and maximum link ratio 0.5 (3). The GAEMR package (www.broadinstitute.org/software/gaemr/) was used to evaluate the contiguity, the completeness and the correctness of the generated assemblies using the genomes and gene annotations of Pb18, Pb03 and Pb01 as references, as well as from closely related *Blastomyces* and *Histoplasma* (4).

Genome annotation: Protein-coding genes were predicted using a combination of gene models from the prediction programs Augustus (5), Genemark-ES (6), GlimmerHMM (7), Genewise (8), and Snap (9), and manual gene revision of flagged calls. The predicted gene sets were then provided as input to EvidenceModeler (EVM; (10)) to obtain the best consensus model for a given locus. The consistency of the gene models was evaluated by examining alignments of protein orthology groups identified using OrthoMCL (11). EVMLite was used to rescue orphan genes not captured in EVM; only those genes with additional evidence such as overlap with Genewise or non-repeat HMMER3 PFAM domains were rescued, as well as non-redundant genes overlapping the OrthoMCL genes in clusters containing 2 or more genomes. Lastly spurious gene models matching repetitive or low-complexity sequences were removed. In order to evaluate the quality of the final predicted gene set we compared with Pb01, Pb03 and Pb18 (12). Each annotated gene was assigned a locus number of the form GX48_##### for PbCnh (PS3) and ACO22_##### for Pb300 (PS4) which serves as a unique identifier within each genome and across assemblies.

The SOAPdenovo assembly of the strain PbCnh with *kmer* value 37 was chosen for the PS3 lineage. The strain Pb300 assembled using a combination of Spades, SSpace and Pilon was selected as the reference for the PS4 lineage. For PbCnh (PS3) a total of 392 scaffolds were assembled in 29.4 Mb, with a scaffold-N50 of 214.2 kb. For Pb300 (PS4) a total of 1,368 scaffolds were assembled also in 29.4 Mb with a scaffold-N50 of 52 kb (**Figure S1A**). The assembly sizes of PbCnh and Pb300 are between the assembly size of the previous sequenced genomes of *P. brasiliensis* from the S1b and PS2 lineages, *i.e.* Pb18 and Pb03 are 29.95 Mb and 29.06 Mb respectively (**Figure S1C**; (12)).

Predicted gene content was highly similar across the *P. brasiliensis* genomes, comparing the new assemblies to the previously sequenced Pb18 (S1b) and Pb03 (PS2). The annotation of the PbCnh and Pb300 resulted in 8,324 and 8,070 predicted protein-coding genes respectively. The total number of genes and associated functional annotation are similar in comparison with Pb18 and Pb03, supporting that the obtained genome assemblies and final gene sets are high quality reference genomes for the PS3 and PS4 lineages (**Figure S1C**). High representation of core eukaryotic genes provides evidence that those genomes are nearly complete; *P. brasiliensis* (PbCnh and Pb300) includes 96-98% of core eukaryotic genes (**Figure S1B**).

Whole genome alignment and Synteny analysis: We performed whole genome comparisons among the five *Paracoccidioides* genomes (Pb01, Pb03, Pb18, PbCnh and Pb300) by sequence alignment and similarity analyses using the nucmer module to align genomes with minimum match length 200, and compute MUMs option, and the mummerplot module to generate similarity plots, both modules part of the MUMmer v3.22 package (13). Syntenies among the genomes at the level of gene order/orientation were assessed with the DAGchainer program (14). The program was used to identify syntenic blocks of at least 4 genes, which were required to be in the same order and orientations in the compared genomes.

In addition, we looked for evidence of aberrant mapping of the paired reads to the Pb18 reference assembly, using BreakDancer 1.1.2 to detect large structural variants (15). In addition to rearrangements found by whole genome alignment and synteny analyses, additional variants including large inversions and deletions were detected (**Data Set 1**). A total of 14 genes fall into these deleted regions, including a protein kinase, a predicted transcription factor, among other unknown predicted genes that are unique in Pb18 (see below).

The *Paracoccidioides* genomes of both species and all lineages are highly conserved in terms of whole genome sequence similarity and gene synteny (**Figure 1**). The genomes of *P. brasiliensis* share an average of 98.5% identity, whereas the more distant *P. lutzii* shares an average of 94.8% with them. Alignments cover nearly the entirety of each assembly; the genomes of PbCnh (PS3) and Pb300 (PS4) share the highest percent aligned (98.9%), followed by PbCnh and Pb18 (98.7%), whereas *P. brasiliensis* PbCnh (PS3) and Pb01 (*Pl*) share the lowest percent of aligned (94.5%) (**Figure S1D**). These syntenic percentages are correlated with the phylogenetic and population structure relationships. To further examine genome organization, we identified syntenic regions of conserved gene order (**Figure S1D**). An average of 6,907 genes were found in syntenic blocks among the *Paracoccidioides* lineages. The percent of genes in syntenic blocks ranged from 75.3% (interspecies, *Pb* vs. *Pl*) to 89.9% (intraspecies, *Pb* vs. *Pb*). By contrast, the *Blastomyces* dimorphic fungus from the Ajellomycetaceae family has only ~69% genes in syntenic blocks between isolates due to the presence of isochore-like structures of GC-poor and GC-rich blocks rarely observed in *Paracoccidioides* (4). Genome structure appears highly conserved among *P. brasiliensis* lineages as well as between *P. brasiliensis* and *P. lutzii*. In both species lineage specific genes are dispersed along each genome and not clustered in specific regions (**Figure S1F**). The presence and location of known virulence factors in *Paracoccidioides* such as the antigens *GP43* and *P27* are highly conserved between *Paracoccidioides* spp. (**Figure S1F**).

**Gene conservation in *Paracoccidioides***

The completion of reference genomes for the PS3 and PS4 lineage allowed us to re-examine gene content differences across *Paracoccidioides*. A total of 6,670 core ortholog clusters had representative genes from all five genomes. A larger set of 8,432 ortholog clusters are conserved in at least two *Paracoccidioides* genomes. From these ortholog clusters an average of 280 genes were specific to each of the *P. brasiliensis* lineages, and 1,340 genes were species-specific to *P. lutzii*. The higher gene number correlates with the genome expansion of 3.5 Mb in *P. lutzii* strain Pb01.

Comparing the *P. brasiliensis* lineages, we found 720 ortholog groups in at least two strains and 459 ortholog groups that were present in all *P. brasiliensis* strains but absent in *P. lutzii*. We did not find any significant enrichment of functional categories (PFAM, GO or KEGG terms) among *P. lutzii* and *P. brasiliensis* or among their lineages, which suggests that the phenotypic differences between *P. lutzii* and *P. brasiliensis*, and between the lineages are not due to large protein family expansion or contraction. However, unique genes could lead to different phenotypic differences observed in each lineage, as well as genes duplicated in *P. brasiliensis*, but not in *P. lutzii* and *vice versa* (**Data Set S1 Tab4**). Among the unique genes we identified, there are several protein kinases, transcription factors, and transporters. In *P. lutzii* there are several abhydrolases, glycosyl hydrolases, peptidases (M24, C12), and methyltransferases. In *P. brasiliensis* there are actin genes, transporters, aspartyl proteases, peptidases (M16, M28), and several transcription factors. Some of these unique genes, such as the aspartyl proteases and other peptidases, are known to be involved in pathogenesis in other fungi. This suggests that these unique proteins may provide a more diverse repertoire to each *P. lutzii* or *P. brasiliensis* lineage, enabling the fungus different mechanisms and strategies to produce infection and disease. However, additional phenotypic differences may be due to differences in intergenic regions such as promoter regions. Repetitive elements could also result in gene inactivation or disruption, which may not have been identified by the gene-calling pipeline, and variants within gene regulatory elements may also confer important phenotypic differences among the lineages.

**Candidate genes with experimental evidence of a role in virulence and pathogenicity**

Genes located in these regions were not enriched for any specific function and encompassed different cellular functions including transport, transcription regulation, oxidative stress and proteolysis (**Data Set S1 Tab2**). However, interestingly, 35.4% of the genes in high diversity regions have evidence of positive selection, including the antigenic glucan 1,3-beta-glucosidase (*GP43*, PADG_07615) (16), the antigenic GPI-protein (*PGA1*, PADG_02460) (17), other secreted proteins (e.g. PADG_00954, PADG_05055, PADG_02535) and proteases (e.g. PADG_05820, PADG_04167, PADG_06167, PADG_07460) (**Data Set S1 Tab2**). Some of these genes have experimental evidence of a role in virulence and pathogenicity. For example, the ortholog of the secreted protein PADG_05055 that is in a high nucleotide diversity region (significant *pi* and *td* in S1a), *d_N_/d_S_* > 1, and significantly low F_ST_ region, was found to be more highly transcribed in *Blastomyces* during host-pathogen interaction (4). The secreted protein PADG_02535 that is in a high nucleotide diversity region (significant *pi* and *td* in PS3), *d_N_/d_S_* > 1, and significantly low F_ST_ region was identified in extracellular vesicles of *Paracoccidioides* (18), transcriptome of its parasitic phase (Felipe et al., 2005), and its ortholog in *Blastomyces* was more highly expressed during host-pathogen interaction (4). Two aminopeptidases (PADG_05820, PADG_07460) one in a high nucleotide diversity region (significant *pi* in S1b) and the other in a high nucleotide diversity region (significant *pi* and *td* in S1b/PS2), low F_ST_ region, were found to be upregulated in *Paracoccidioides* during macrophage infection (19).

We found notable variation in other genes that could result in differences in *Paracoccidioides* virulence and survival. For example, the purine nucleoside phosphorylase (PADG_08066) is in a high nucleotide diversity region (significant *pi* in S1b, S1a), and was found upregulated in *Paracoccidioides* during macrophage infection (19). The succinate dehydrogenase (PADG_07470) is in a high nucleotide diversity region in *Paracoccidioides* (significant *pi* in S1a), and the amino acid permease (PADG_07440) is in a high nucleotide diversity region (significant *pi* and *td* in PS2/PS3, low F_ST_ region), and the orthologs of both genes in *Blastomyces* were upexpressed during host-pathogen interaction (4). Other genes that fall into these categories and with evidence in the literature for relation with virulence are listed in **Table 2**. In addition, genes for which we do not list experimental evidence are good candidates for experimental analyses.

**References**

1. **Luo R, Liu B, Xie Y, Li Z, Huang W, Yuan J, others.** 2012. SOAPdenovo2: an empirically improved memory-efficient short-read de novo assembler. Gigascience **1:**18.

2. **Bankevich A, Nurk S, Antipov D, Gurevich AA, Dvorkin M, Kulikov AS, Lesin VM, Nikolenko SI, Pham S, Prjibelski AD, Pyshkin AV, Sirotkin AV, Vyahhi N, Tesler G, Alekseyev MA, Pevzner PA.** 2012. SPAdes: a new genome assembly algorithm and its applications to single-cell sequencing. J Comput Biol **19:**455-477.

3. **Boetzer M, Henkel CV, Jansen HJ, Butler D, Pirovano W.** 2011. Scaffolding pre-assembled contigs using SSPACE. Bioinformatics **27:**578-579.

4. **Munoz JF, Gauthier GM, Desjardins CA, Gallo JE, Holder J, Sullivan TD, Marty AJ, Carmen JC, Chen Z, Ding L, Gujja S, Magrini V, Misas E, Mitreva M, Priest M, Saif S, Whiston EA, Young S, Zeng Q, Goldman WE, Mardis ER, Taylor JW, McEwen JG, Clay OK, Klein BS, Cuomo CA.** 2015. The Dynamic Genome and Transcriptome of the Human Fungal Pathogen *Blastomyces* and Close Relative *Emmonsia*. PLoS Genet **11:**e1005493.

5. **Stanke M, Waack S.** 2003. Gene prediction with a hidden Markov model and a new intron submodel. Bioinformatics **19 Suppl 2:**ii215-225.

6. **Ter-Hovhannisyan V, Lomsadze A, Chernoff YO, Borodovsky M.** 2008. Gene prediction in novel fungal genomes using an ab initio algorithm with unsupervised training. Genome Res **18:**1979-1990.

7. **Majoros WH, Pertea M, Salzberg SL.** 2004. TigrScan and GlimmerHMM: two open source ab initio eukaryotic gene-finders. Bioinformatics **20:**2878-2879.

8. **Birney E, Clamp M, Durbin R.** 2004. GeneWise and Genomewise. Genome Res **14:**988-995.

9. **Korf I.** 2004. Gene finding in novel genomes. BMC Bioinformatics **5:**59.

10. **Haas BJ, Salzberg SL, Zhu W, Pertea M, Allen JE, Orvis J, White O, Buell CR, Wortman JR.** 2008. Automated eukaryotic gene structure annotation using EVidenceModeler and the Program to Assemble Spliced Alignments. Genome Biol **9:**R7.

11. **Li L, Stoeckert CJ, Jr., Roos DS.** 2003. OrthoMCL: identification of ortholog groups for eukaryotic genomes. Genome Res **13:**2178-2189.

12. **Munoz JF, Gallo JE, Misas E, Priest M, Imamovic A, Young S, Zeng Q, Clay OK, McEwen JG, Cuomo CA.** 2014. Genome update of the dimorphic human pathogenic fungi causing paracoccidioidomycosis. PLoS Negl Trop Dis **8:**e3348.

13. **Kurtz S, Phillippy A, Delcher AL, Smoot M, Shumway M, Antonescu C, Salzberg SL.** 2004. Versatile and open software for comparing large genomes. Genome Biol **5:**R12.

14. **Haas BJ, Delcher AL, Wortman JR, Salzberg SL.** 2004. DAGchainer: a tool for mining segmental genome duplications and synteny. Bioinformatics **20:**3643-3646.

15. **Chen K, Wallis JW, McLellan MD, Larson DE, Kalicki JM, Pohl CS, McGrath SD, Wendl MC, Zhang Q, Locke DP, Shi X, Fulton RS, Ley TJ, Wilson RK, Ding L, Mardis ER.** 2009. BreakDancer: an algorithm for high-resolution mapping of genomic structural variation. Nat Methods **6:**677-681.

16. **Puccia R, Travassos LR.** 1991. 43-kilodalton glycoprotein from *Paracoccidioides brasiliensis*: immunochemical reactions with sera from patients with paracoccidioidomycosis, histoplasmosis, or Jorge Lobo's disease. J Clin Microbiol **29:**1610-1615.

17. **Valim CX, Basso LR, Jr., dos Reis Almeida FB, Reis TF, Damasio AR, Arruda LK, Martinez R, Roque-Barreira MC, Oliver C, Jamur MC, Coelho PS.** 2012. Characterization of PbPga1, an antigenic GPI-protein in the pathogenic fungus *Paracoccidioides brasiliensis*. PLoS One **7:**e44792.

18. **Peres da Silva R, Heiss C, Black I, Azadi P, Gerlach JQ, Travassos LR, Joshi L, Kilcoyne M, Puccia R.** 2015. Extracellular vesicles from *Paracoccidioides* pathogenic species transport polysaccharide and expose ligands for DC-SIGN receptors. Sci Rep **5:**14213.

19. **Parente-Rocha JA, Parente AF, Baeza LC, Bonfim SM, Hernandez O, McEwen JG, Bailao AM, Taborda CP, Borges CL, Soares CM.** 2015. Macrophage Interaction with *Paracoccidioides brasiliensis* Yeast Cells Modulates Fungal Metabolism and Generates a Response to Oxidative Stress. PLoS One **10:**e0137619.
