# Supplementary material for: Genome Diversity, Recombination, and Virulence across the Major Lineages of Paracoccidioides
Source: mSphere. 2016 Sep 28;1(5):e00213-16. doi: 10.1128/mSphere.00213-16 (PMC5040785; doi:10.1128/mSphere.00213-16)

A

Basic Assembly Stats	PbCNH	Pb300
Contigs	1,382	2,389
Max Contig (kb)	432	127
Mean Contig (kb)	21	12
Contig N50 (kb)	46	25
Contig N90 (kb)	12	6
Total Contig Length (Mb)	29.3	29.3
Assembly GC	43.9	44.3
Scaffolds	392	1,368
Max Scaffold (kb)	718	342
Mean Scaffold (kb)	75	22
Scaffold N50 (kb)	214	52
Scaffold N90 (kb)	47	10
Total Scaffold Length (Mb)	29.4	29.4

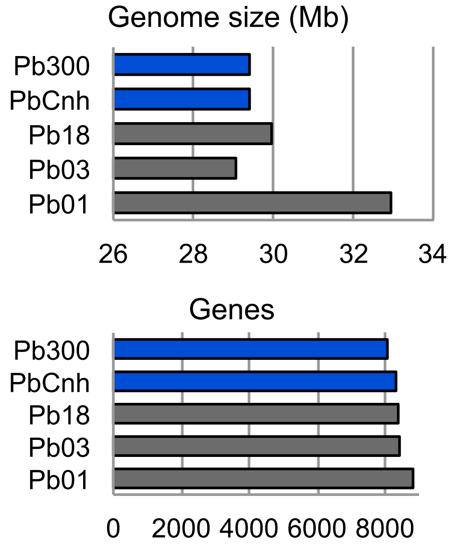

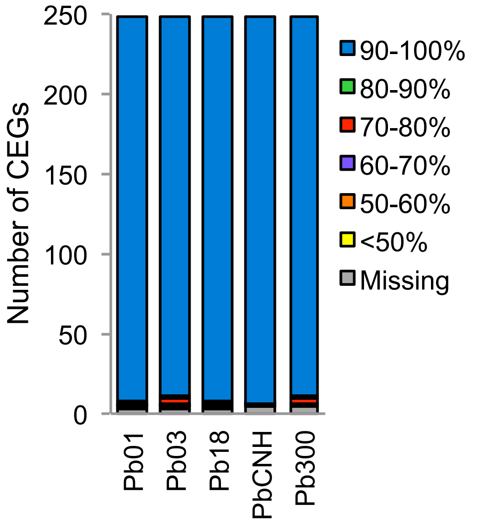

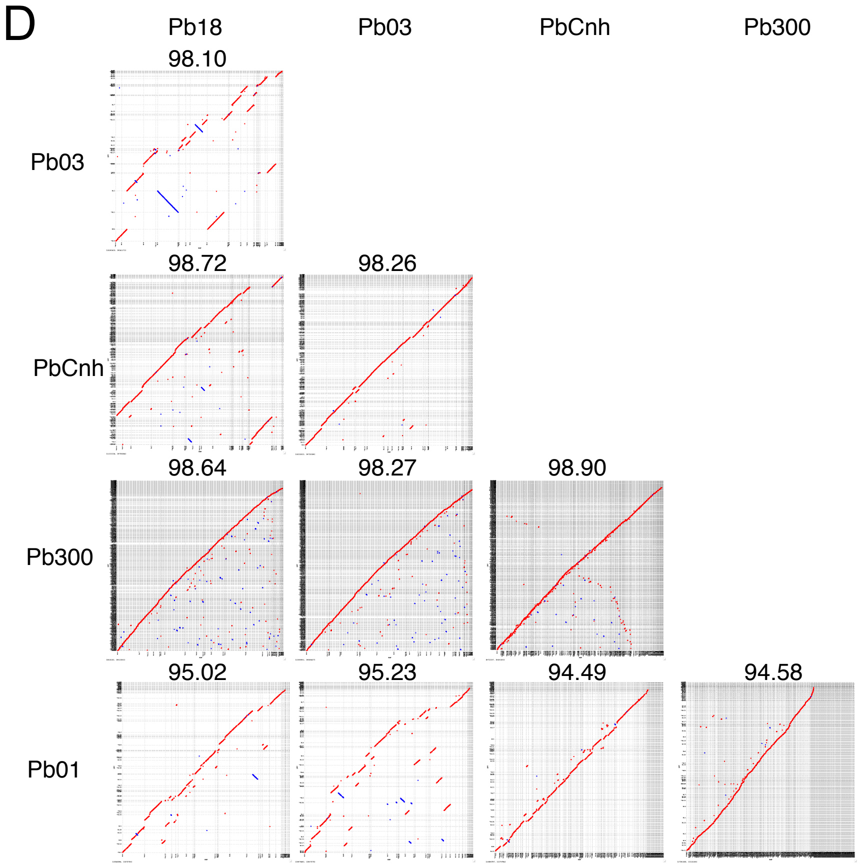

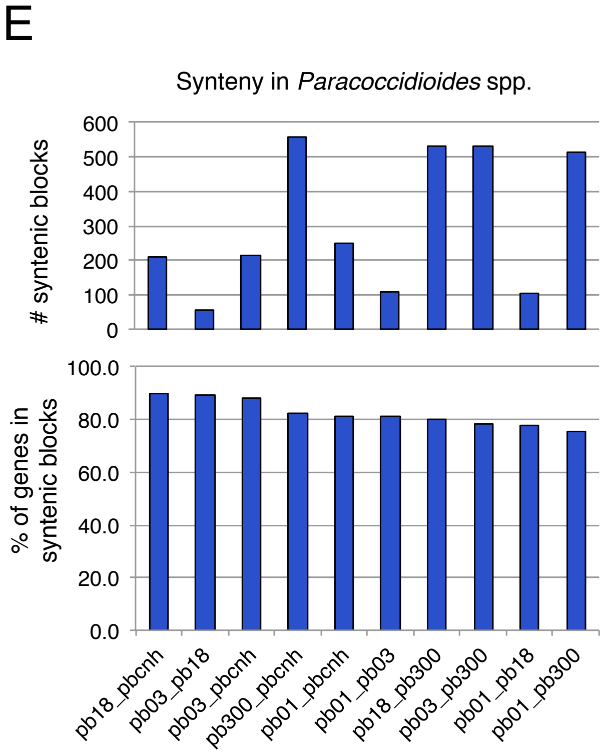

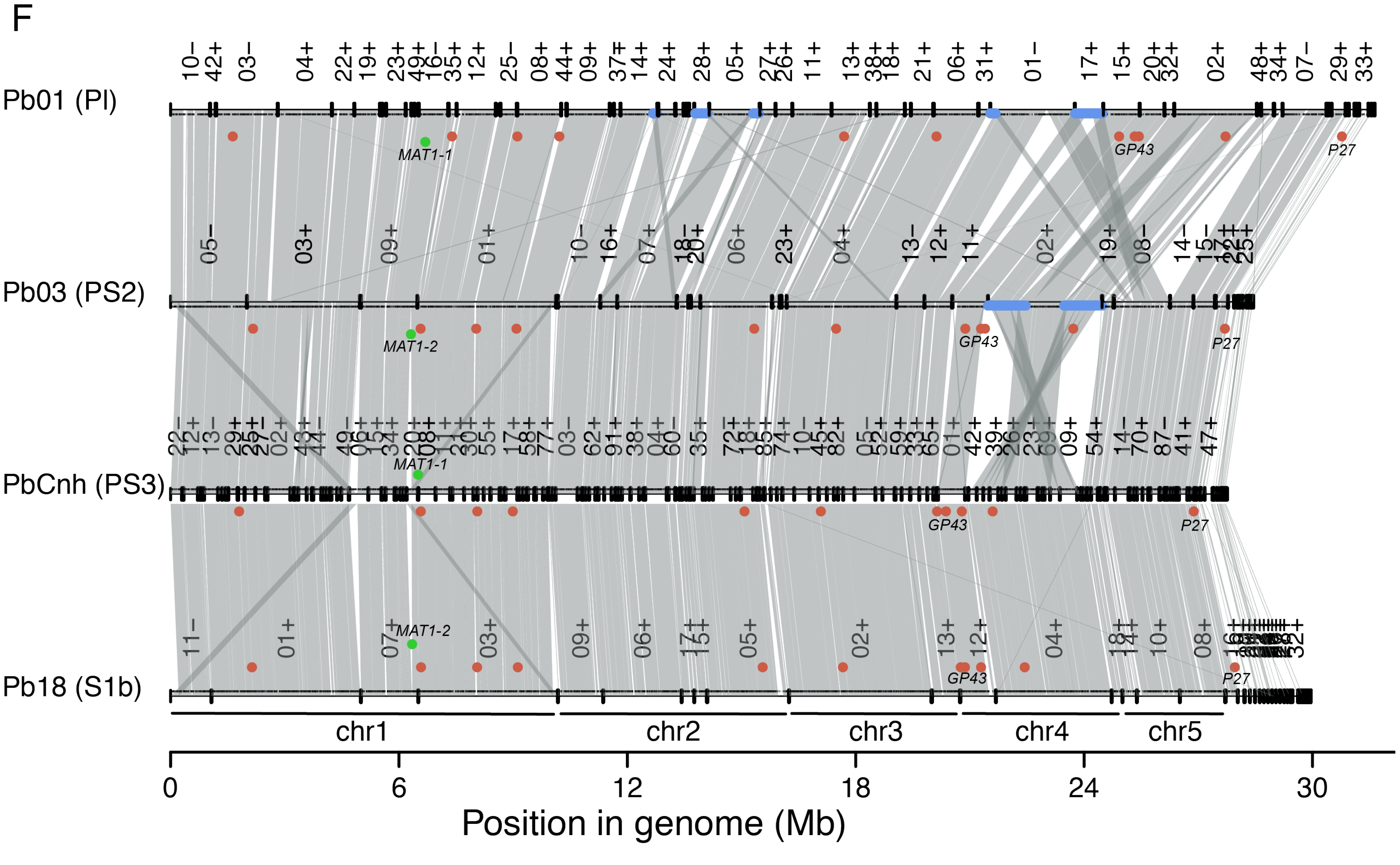

Supplement: Figure S1 [file sph005162156sf2.pdf]
