# Supplementary figures and images for: Genome Diversity, Recombination, and Virulence across the Major Lineages of Paracoccidioides
Source: mSphere. 2016 Sep 28;1(5):e00213-16. doi: 10.1128/mSphere.00213-16 (PMC5040785; doi:10.1128/mSphere.00213-16)

**A**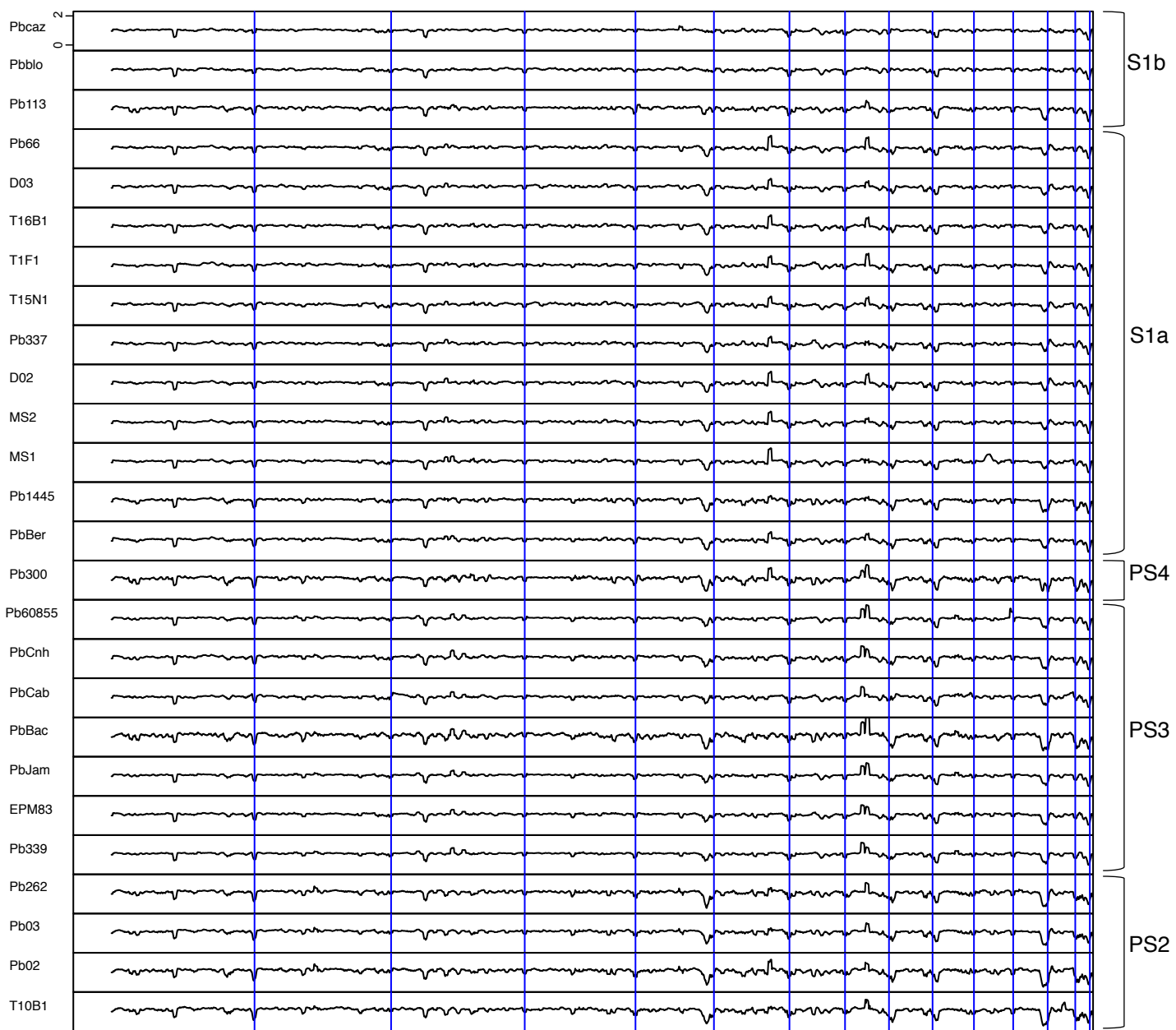**B**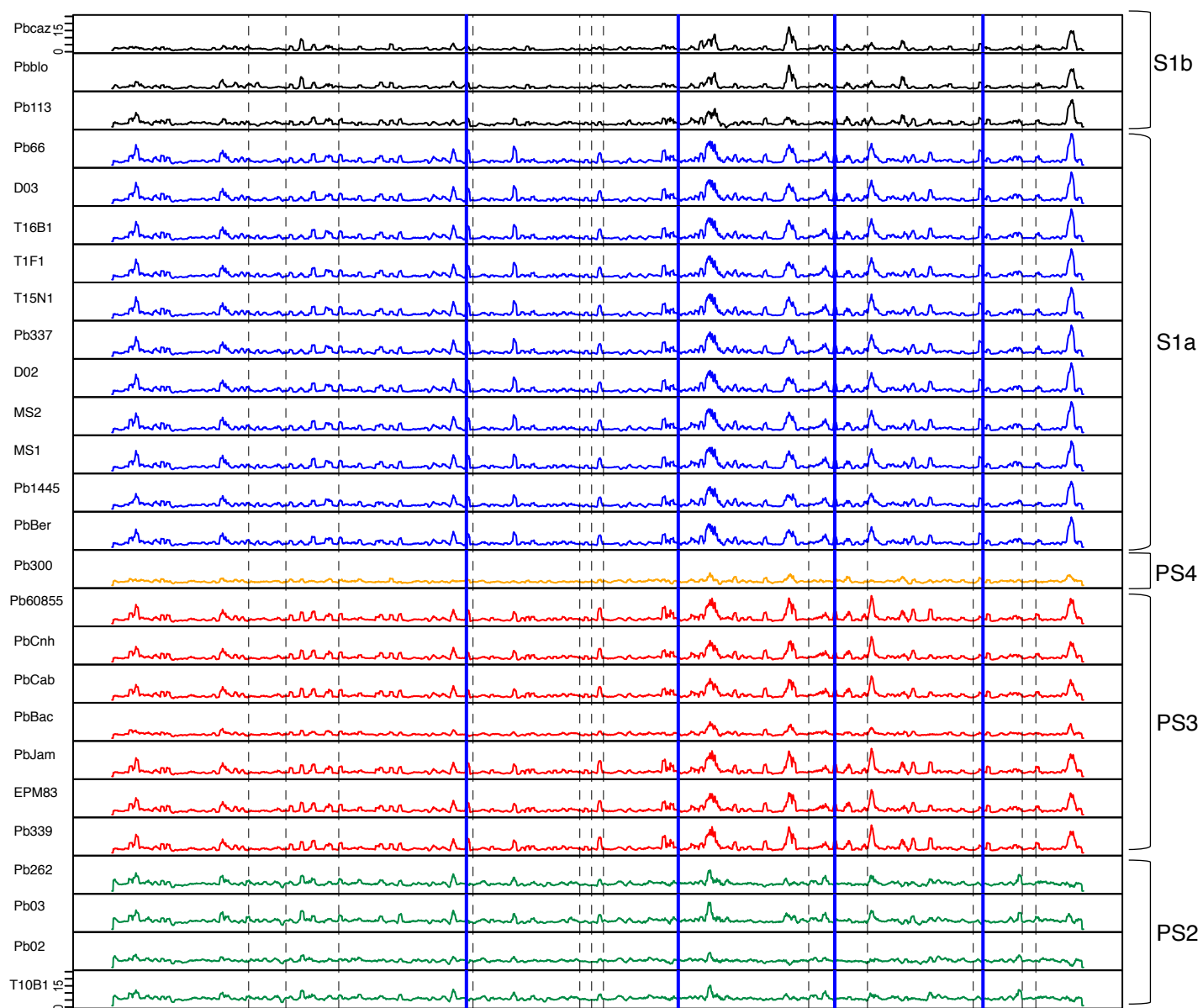

Supplement: Figure S2 [file sph005162156sf3.pdf]

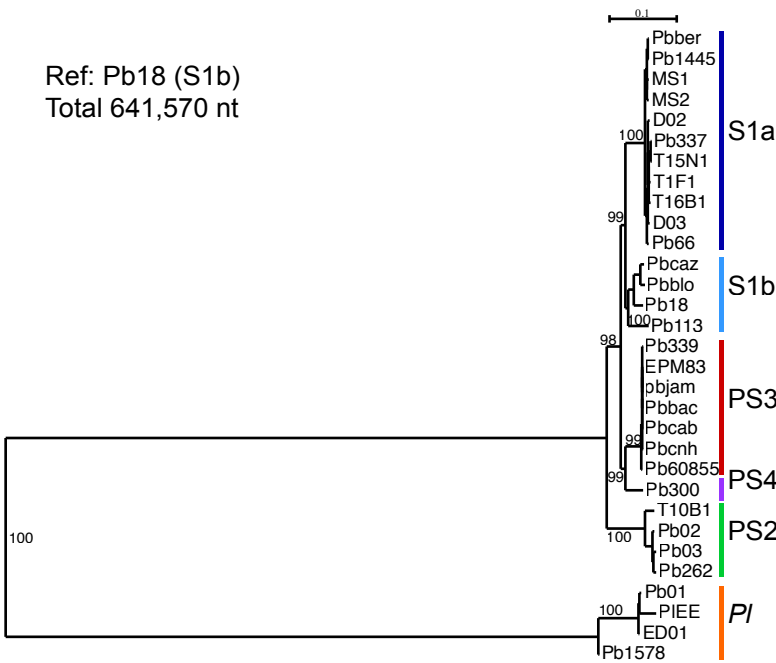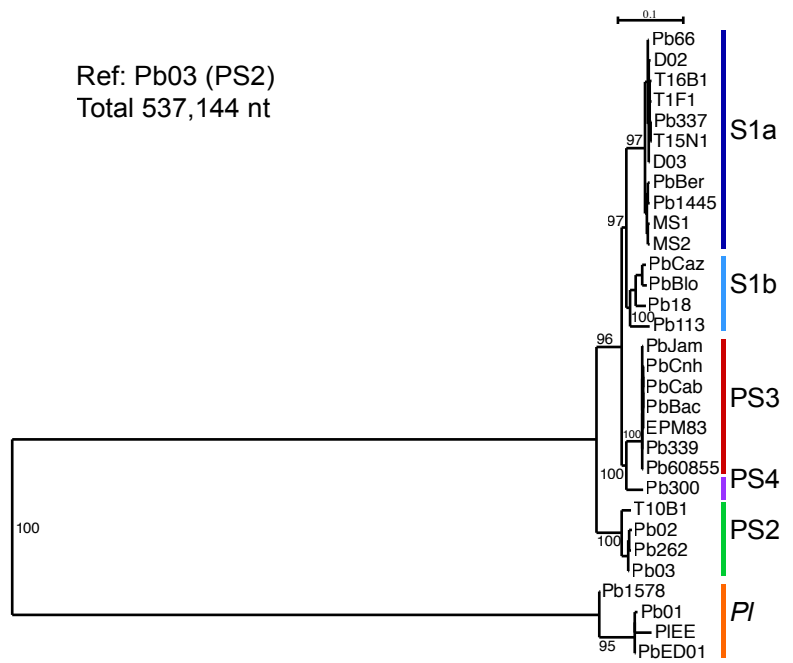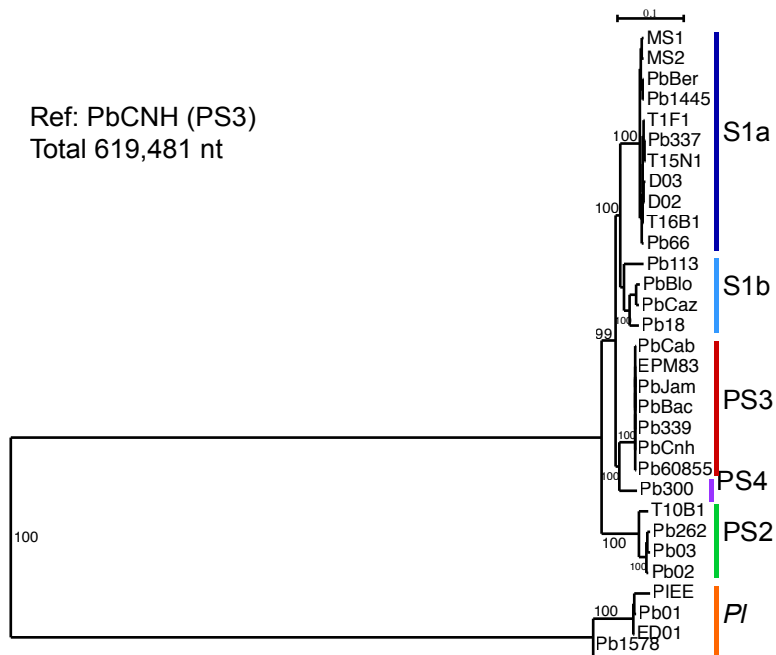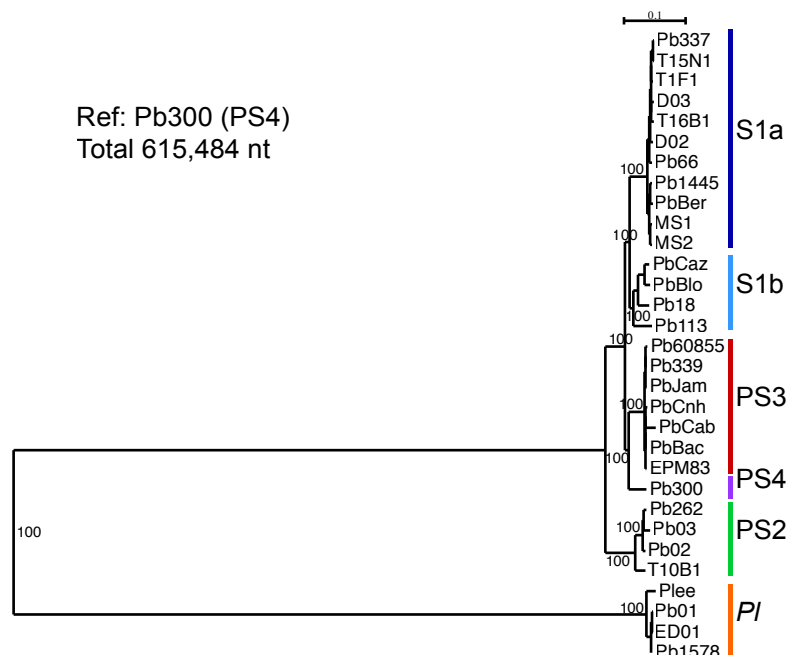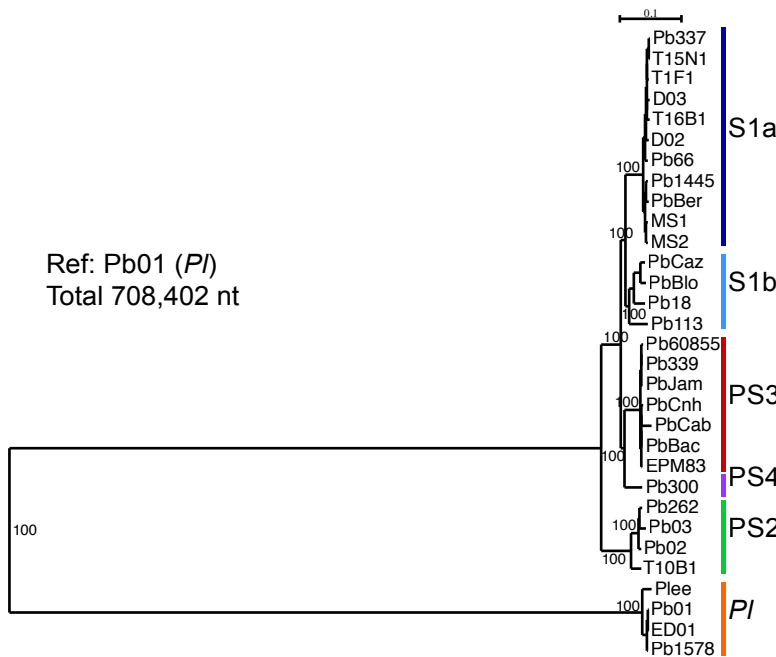

Supplement: Figure S3 [file sph005162156sf4.pdf]

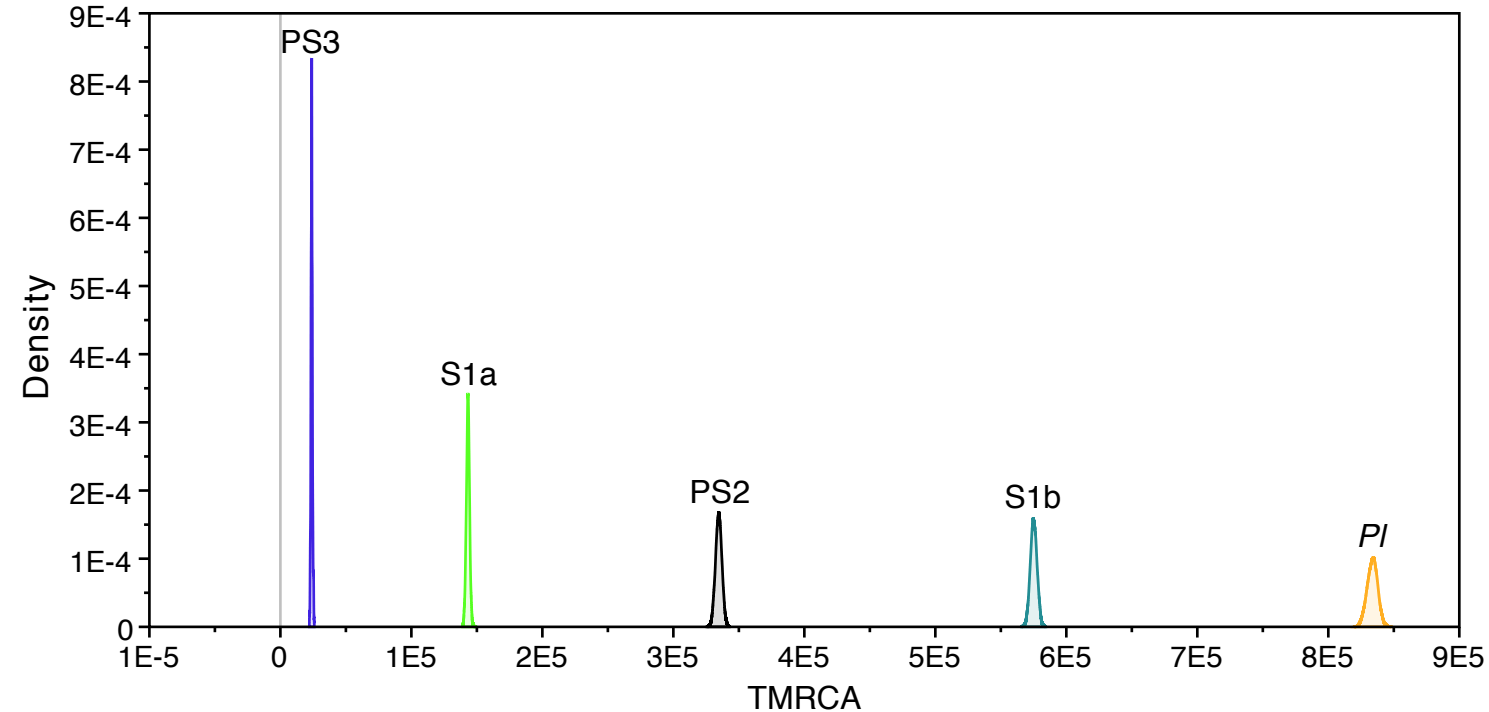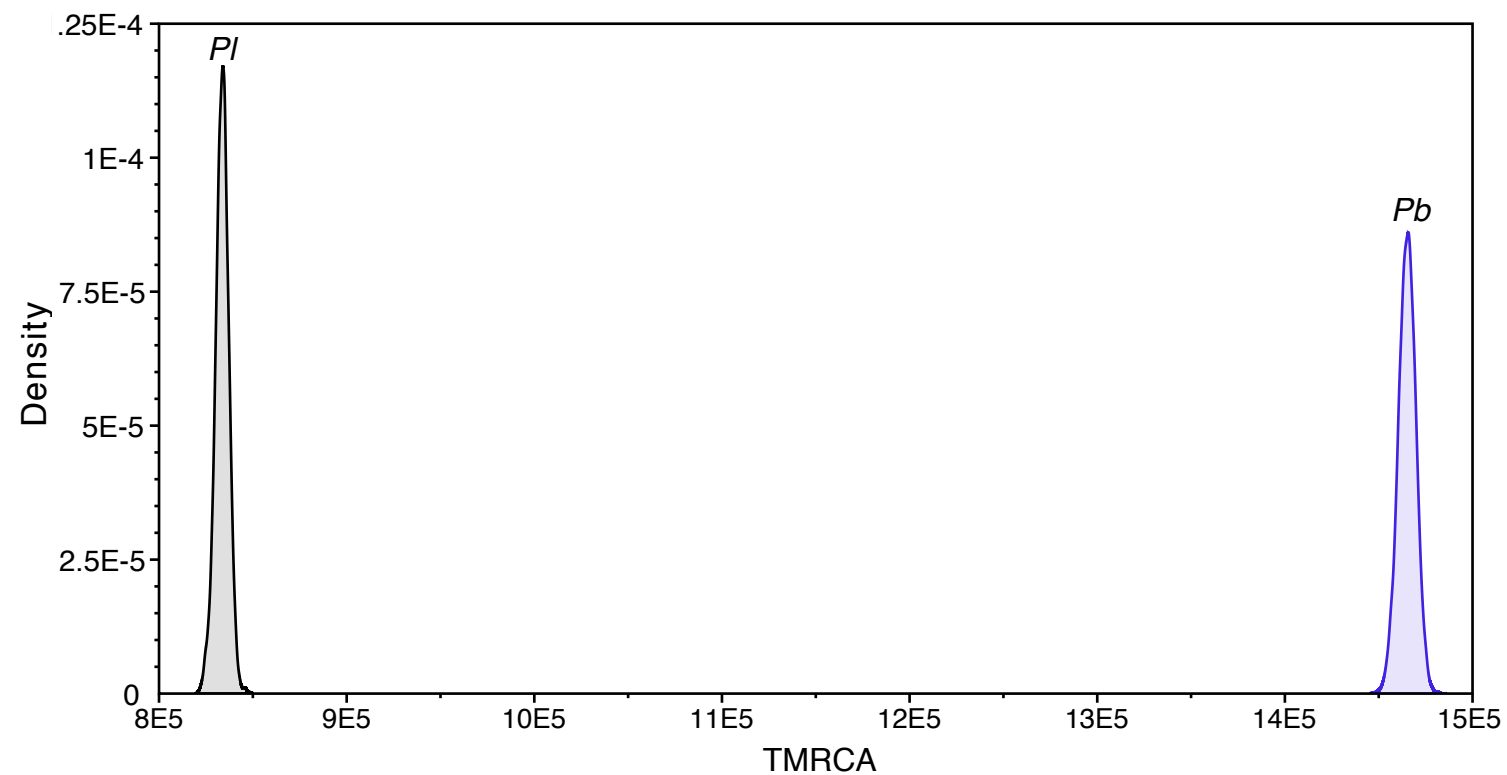

|       | TMRCA      | stderr of mean | stdev   |
|-------|------------|----------------|---------|
| Pb-PI | $2.24E+07$ | 841.7          | 63080.2 |
| PI    | $8.34E+05$ | 264.5          | 3575.5  |
| Pb    | $1.47E+06$ | 283.8          | 4624.5  |
| S1a   | $1.43E+05$ | 47.4           | 1225.5  |
| S1b   | $5.75E+05$ | 91.1           | 2500.6  |
| PS2   | $3.35E+05$ | 105.8          | 2364.4  |
| PS3   | 23909.7158 | 38.5           | 582.0   |

Supplement: Figure S4 [file sph005162156sf5.pdf]

A

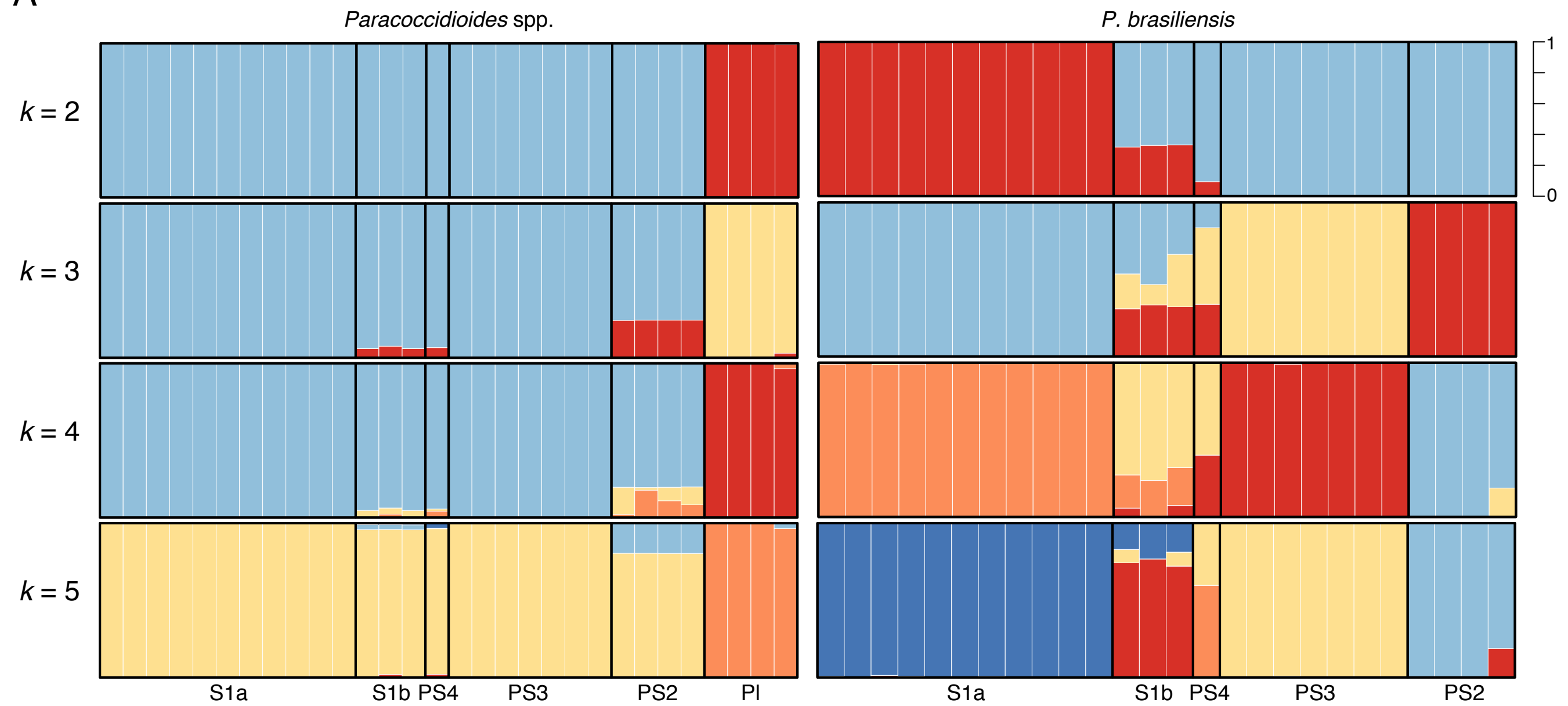

B

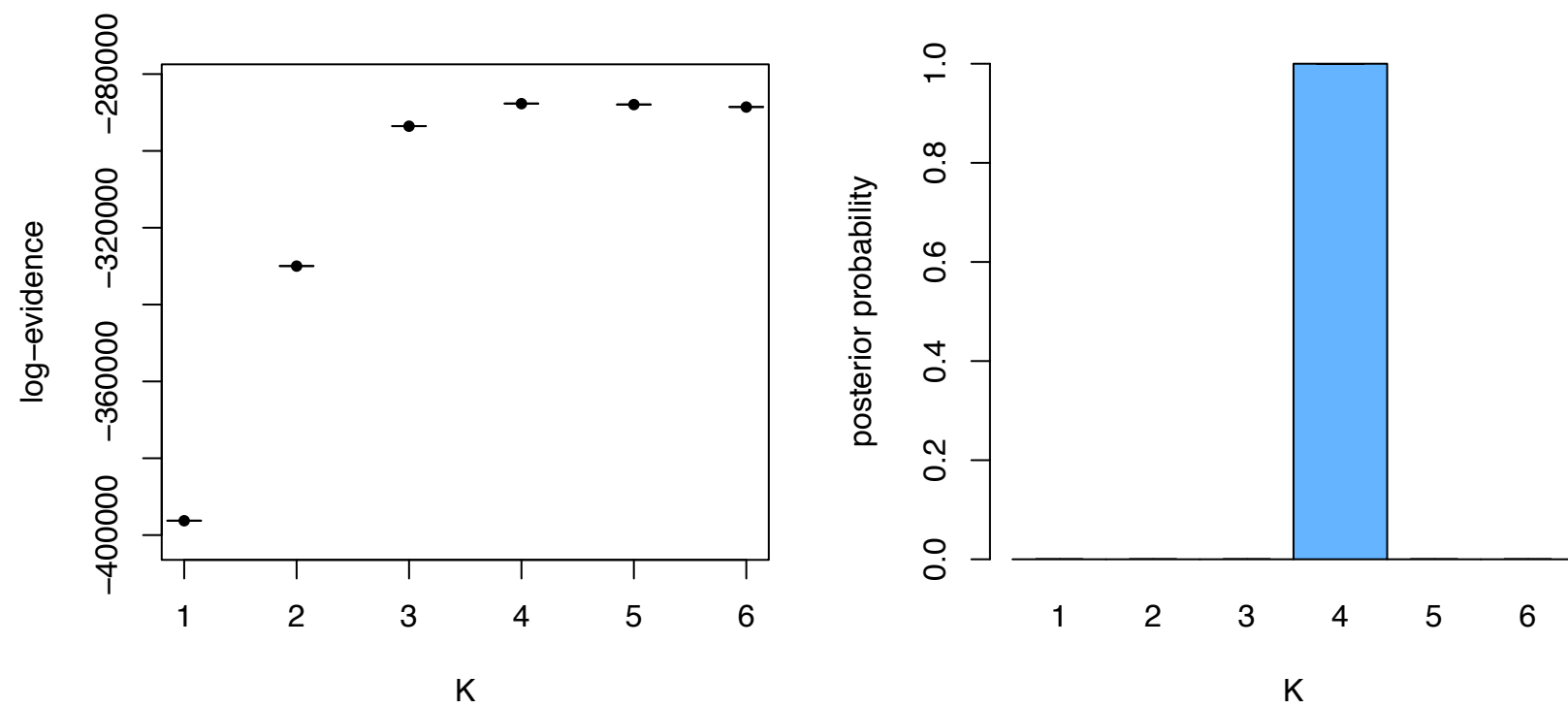

Supplement: Figure S5 [file sph005162156sf6.pdf]

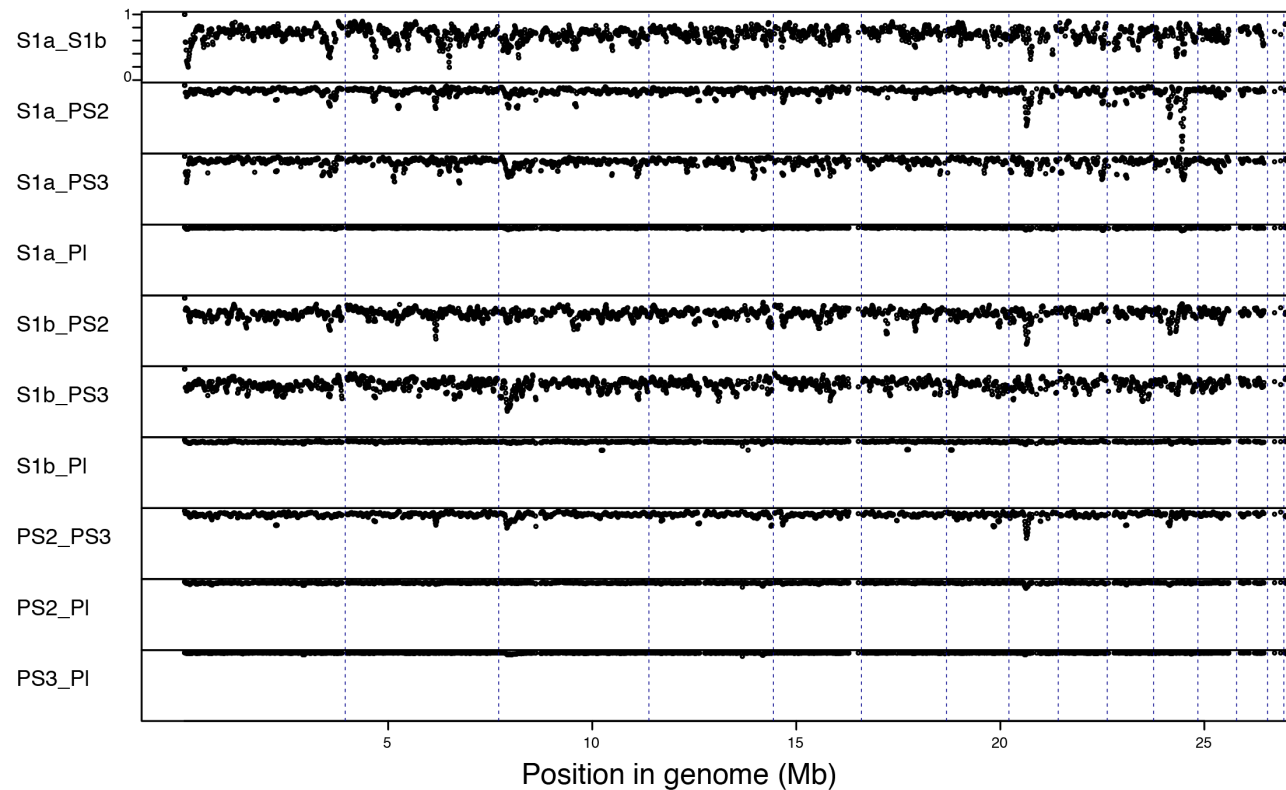

Supplement: Figure S7 [file sph005162156sf8.pdf]
