# Supplementary material for: Genome Diversity, Recombination, and Virulence across the Major Lineages of Paracoccidioides
Source: mSphere. 2016 Sep 28;1(5):e00213-16. doi: 10.1128/mSphere.00213-16 (PMC5040785; doi:10.1128/mSphere.00213-16)
Supplement: Figure S6 [file sph005162156sf7.pdf]

lineage	strain	mat1-1 (alpha)	mat1-2 (HMG)	mat1-1 (alpha)	mat1-2 (HMG)
s1a	pb1445	•		2350	1
s1a	pbber	•		2350	1
s1a	d02	•		2350	1
s1a	t1f1	•		2350	1
s1a	t16b1	•		2350	1
s1a	ms1		•	0	1100
s1a	d03		•	0	1100
s1a	ms2		•	0	1100
s1a	pb337		•	0	1100
s1a	pb66		•	0	1100
s1a	t15n1		•	0	1100
s1b	pbcaz	•		2350	1
s1b	pb113	•		2350	1
s1b	pb18		•	0	1100
s1b	pbblo		•	0	1100
ps4	pb300		•	0	1100
ps3	pb339	•		2349	1
ps3	pbbac	•		2349	1
ps3	pbcab	•		2349	1
ps3	pbcnh	•		2349	1
ps3	epm83		•	0	1100
ps3	pb60855		•	0	1100
ps3	pbjam		•	0	1100
ps2	pb02	•		2350	0
ps2	pb03		•	0	1100
ps2	pb262		•	0	1100
ps2	t10b1		•	0	1100
pl	pb01	•		1988	0
pl	pb1578	•		1664	0
pl	ed01	•		1632	0
pl	plee		•	0	1102
	S1a	5	6		
	S1b	2	2		
	PS4	0	1		
	PS3	4	3		
	PS2	1	3		
	Pl	3	1		
	**Total**	**15**	**16**		

Annotation	mating type alpha box protein (MAT1-1)	mating type HMG box protein (MAT1-2)
Pb01 (*Pl*)	**PAAG_05873**	
Pb03 (PS2)		**PABG_05778**
Pb18 (S1b)		**PADG_06118**
PbCnh (PS3)	**GX48_02691**	
Pb300 (PS4)		**ACO22_07838**
